# Robust Wannierization including magnetization and spin-orbit coupling via projectability disentanglement

**DOI:** 10.1038/s41524-025-01835-8

**Published:** 2025-11-21

**Authors:** Yuhao Jiang, Junfeng Qiao, Nataliya Paulish, Weisheng Zhao, Nicola Marzari, Giovanni Pizzi

**Affiliations:** 1 PSI Center for Scientific Computing, Theory and Data, Villigen PSI, Switzerland; 2 Fert Beijing Institute, School of Integrated Circuit Science and Engineering, Beihang University, Beijing, China; 3 Theory and Simulations of Materials (THEOS), and National Centre for Computational Design and Discovery of Novel Materials (MARVEL), École Polytechnique Fédérale de Lausanne, Lausanne, Switzerland; 4 National Key Lab of Spintronics, Institute of International Innovation, Beihang University, Hangzhou, China

**Keywords:** Materials science, Physics

## Abstract

Maximally-localized Wannier functions (MLWFs) are widely employed as an essential tool for calculating the physical properties of materials due to their localized nature and computational efficiency. Projectability-disentangled Wannier functions (PDWFs) have recently emerged as a reliable and efficient approach for automatically constructing MLWFs that span both occupied and lowest unoccupied bands. Here, we extend the applicability of PDWFs to magnetic systems and/or those including spin-orbit coupling, and implement such extensions in automated workflows. Furthermore, we enhance the robustness and reliability of constructing PDWFs by defining an extended protocol that automatically expands the projectors manifold, when required, by introducing additional appropriate hydrogenic atomic orbitals. We benchmark our extended protocol on a set of 200 chemically diverse materials, as well as on the 40 systems with the largest band distance obtained with the standard PDWF approach, showing that on our test set the present approach delivers a success rate of over 98% in obtaining accurate Wannier-function interpolations, defined as an average band distance below 20 meV between the DFT and Wannier-interpolated bands, up to 2 eV above the Fermi level for metals or above the conduction band minimum for insulators (and a 100% success rate when including only bands up to 1 eV above these values).

## Introduction

In materials science calculations, density-functional theory (DFT)^1^ is nowadays an established and highly versatile method, widely used for calculating various physical properties of extended systems and molecules. However, to accurately capture several properties of crystalline materials, such as for instance the anomalous Hall effect^2^ or the spin Hall effect^3,4^, calculations on a very dense *k*-point mesh in the Brillouin zone are required, often involving even millions of *k*-points^5,6^ to achieve convergence. Solving the Kohn-Sham equations^7^ independently for such an extensive number of *k*-points incurs significant computational cost.

An alternative approach is to construct a tight-binding model using a real-space basis of Wannier functions (WFs)^8^. Maximally localized Wannier functions (MLWFs)^9^ can be efficiently obtained from Bloch functions via a unitary transformation between the Bloch wavefunctions at every *k*-point, and then a Fourier transformation to real space. These functions allow for efficient interpolation of wavefunctions onto arbitrary *k*-point meshes at a low computational cost. This approach has been widely adopted in particular for calculating physical quantities requiring extensive *k*-point integration^10^, such as the density of states (DOS)^11^, Boltzmann transport^12^, anomalous Hall effect^13^, orbital magnetic moments^14^, and the spin Hall effect^15,16^.

WFs $\left\vert {w}_{n{\bf{R}}}\right\rangle$ are obtained via a Fourier transformation of the Bloch states $\left\vert {\psi }_{n{\bf{k}}}\right\rangle$ associated to the same band *n*,

1 $$\left\vert {w}_{n{\bf{R}}}\right\rangle =\frac{V}{{(2\pi )}^{3}}{\int}_{\!\!BZ}d{\bf{k}}{e}^{-i{\bf{kR}}}\left\vert {\psi }_{n{\bf{k}}}\right\rangle ,$$

where *V* is the volume of the primitive cell, and **k** and **R** are the Bloch quasi-momentum in the BZ and a real-space lattice vector, respectively. However, there is a gauge freedom of the Bloch functions where each Bloch state can be multiplied by a phase factor ${e}^{i{\phi }_{n}({\bf{k}})}$, dependent both on *n* and **k**, without changing the Hilbert space but changing the shape of the WFs (and in particular their localization in real space). MLWFs utilize such a gauge freedom to obtain the most localized WFs^9^ by minimizing a quadratic spread functional

2 $$\Omega =\mathop{\sum }\limits_{n=1}^{J}[\langle {w}_{n{\boldsymbol{0}}}| {{\bf{r}}}^{2}| {w}_{n{\boldsymbol{0}}}\rangle -| \langle {w}_{n{\boldsymbol{0}}}| {\bf{r}}| {w}_{n{\boldsymbol{0}}}\rangle {| }^{2}],$$

where *J* is the number of target Wannier bands. For multi-band systems, the gauge freedom is further generalized to allow mixing between different bands, rather than being limited to a simple exponential phase factor. This freedom can be encoded in a set of unitary matrices *U*_*mn***k**_, so that MLWFs can be expressed as

3 $$\left\vert {w}_{n{\bf{R}}}\right\rangle =\frac{V}{{(2\pi )}^{3}}{\int}_{\!\!BZ}d{\bf{k}}{e}^{-i{\bf{kR}}}\mathop{\sum }\limits_{m=1}^{{J}_{{\bf{k}}}}\left\vert {\psi }_{m{\bf{k}}}\right\rangle {U}_{mn{\bf{k}}}.$$

For an isolated set of bands, such as the valence bands of semiconductors or insulators, *J*_**k**_ is a constant that equals to *J*, and the *U*_*mn***k**_ are unitary square matrices. For metallic systems, where the energy bands are entangled, one should select more bands and perform a disentanglement procedure^17^. The number *J*_**k**_ of selected bands is *k*-dependent, and *U*_*mn***k**_ are semi-unitary rectangular matrices. Then, the WFs can be transformed back to Bloch states ${\psi }_{n\tilde{{\bf{k}}}}$ on any other *k*-point $\tilde{{\bf{k}}}$ through the inverse Fourier transform

4 $$\left\vert {\psi }_{n{\tilde{\bf{k}}}}\right\rangle =\sum _{{\bf{R}}}{e}^{i{\tilde{\bf{k}}}{\bf{R}}}\left\vert {w}_{n{\bf{R}}}\right\rangle$$

and similarly we can interpolate operators on denser *k*-point meshes in reciprocal space, thereby enabling accurate and efficient computations of various physical properties^11–16^.

In practice, the algorithm to obtain MLWFs by minimizing Eq. (2) is typically implemented via an iterative algorithm, for which an adequately localized initial guess must be provided. This initial guess needs to be sufficiently close to the final Wannier functions to achieve convergence and, until very recently, its selection required physical intuition. One common approach is to project Bloch functions onto hydrogenic wave functions $\left\vert {g}_{n}\right\rangle$^9^ indexed by *n*, to obtain

5 $$\left\vert {\phi }_{n{\bf{k}}}\right\rangle =\mathop{\sum }\limits_{m=1}^{{J}_{{\bf{k}}}}\left\vert {\psi }_{m{\bf{k}}}\right\rangle \langle {\psi }_{m{\bf{k}}}| {g}_{n}\rangle .$$

Since the *U*_*mn***k**_ should be unitary matrices, the projection matrices *A*_*mn***k**_ = 〈*ψ*_*m***k**_∣*g*_*n*_〉 are further orthonormalized through the Löwdin orthonormalization algorithm^18^ to obtain the starting *U*_*mn***k**_ matrices for the minimization procedure. Furthermore, for entangled bands the conventional approach [that we label as energy disentanglement (ED)] is to set an energy window to select the disentanglement manifold^17^. An outer window is first defined, including all Bloch states that can be linearly combined to obtain a smaller disentangled manifold. A smaller inner window is then often also used to define frozen states, which are kept unchanged during the disentanglement process. However, this requires manual setting of input parameters, such as the number of bands and target MLWFs, and the parameters determining the shape of hydrogenic projectors, which limits its integration into high-throughput (HT) calculations. Recently, new algorithms have been proposed to address the challenges associated with HT computations^19–22^. Among these, projectability disentanglement (PD)^21^ emerged an efficient and accurate algorithm that can be easily automated. The PD method uses a criterion based on the value of the projectability of each state onto a set of localized pseudo-atomic orbitals (PAOs)^23,24^ to determine the selection of bands used to construct the initial guess. Typically, PAOs are extracted from the pseudopotentials used in DFT calculations. In this context, the value of the projectability *p*_*m***k**_ refers to the projection of the Bloch wave function $\left\vert {\psi }_{m{\bf{k}}}\right\rangle$ onto the PAOs $\left\vert {g}_{n}^{PAO}\right\rangle$, as expressed by

6 $${p}_{m{\bf{k}}}=\sum _{n}\langle {\psi }_{m{\bf{k}}}| {g}_{n}^{PAO}\rangle \langle {g}_{n}^{PAO}| {\psi }_{m{\bf{k}}}\rangle .$$

The main idea of the PD method can be summarized as follows: states with projectability *p*_*m***k**_ ≈ 1 are kept unchanged (in such a case, the set of projectors $\left\vert {g}_{n}\right\rangle$ is an almost complete set for the Bloch state $\left\vert {\psi }_{m{\bf{k}}}\right\rangle$); states with projectability *p*_*m***k**_ ≈ 0 can instead be neglected, as they are essentially not included in the space spanned by the projectors. The remaining states are instead combined as prescribed by the usual disentanglement procedure mentioned above^17^, with the initial guess set as in Eq. (5), to construct the disentangled manifold. We note that other approaches based on the projectability on PAOs have been proposed in the literature^24^. Nevertheless we stress that, in the PDWF method, the PAOs are only used as physically inspired initial guesses for the Wannierization procedure. Therefore, the final basis set used in our calculations is formed by the final MLWFs, which often closely resemble—but are different from—the initial PAOs from the pseudopotential files.

HT calculations based on PDWFs have demonstrated that this method can efficiently produce highly accurate tight-binding (TB) models^21^. Current automated PDWF implementations have only been performed on spin-unpolarized systems, without considering spin degrees of freedom needed to describe, e.g., ferromagnetic, antiferromagnetic^25,26^ and ferrimagnetic^27^ structures. Furthermore, when relativistic effects are also taken into account by introducing spin-orbit coupling (SOC), higher-order magnetic interactions^28,29^, topological structures^30,31^, intricate magnetic structures in real space^32^, spin textures in momentums space^33^ and other SOC-dominated physical phenomena can be described. Therefore, in this paper we aim to extend the PDWF approach to magnetic systems and to systems requiring SOC. In doing so, we also extend the PDWF algorithm by defining a protocol to introduce additional projections in the form of hydrogenic atomic orbitals. As a result, we enhance the overall robustness of the Wannierization process, achieving a remarkable success rate of over 98% in obtaining accurate Wannier interpolations for materials in our test set.

## Results

Calculating physical properties via Wannier functions relies on the accuracy of MLWFs and of the corresponding Hamiltonian interpolation. Therefore, after obtaining MLWFs, it is essential to additionally examine the interpolation quality. For instance, a straightforward and intuitive approach is to verify the accuracy by comparing the interpolated bands with the original DFT results^17,20,21^.

To quantitatively measure the quality of the band structures obtained from Wannier interpolation with respect to the DFT band structures, we employ the definitions from ref. ^34^ to compute the average band distance as the root mean square of the band energy difference:

7 $${\eta }_{\nu }=\sqrt{\frac{{\sum }_{n{\bf{k}}}{\tilde{f}}_{n{\bf{k}}}{\left({\epsilon }_{n{\bf{k}}}^{{\rm{DFT}}}-{\epsilon }_{n{\bf{k}}}^{{\rm{Wan}}}\right)}^{2}}{{\sum }_{n{\bf{k}}}{\tilde{f}}_{n{\bf{k}}}}}$$

and the maximum band distance for any band and *k*-point in the Brillouin zone (BZ):

8 $${\eta }_{\nu }^{max}=\mathop{\max }\limits_{n{\bf{k}}}\left({\tilde{f}}_{n{\bf{k}}}| {\epsilon }_{n{\bf{k}}}^{{\rm{DFT}}}-{\epsilon }_{n{\bf{k}}}^{{\rm{Wan}}}| \right),$$

where ${\tilde{f}}_{n{\bf{k}}}=\sqrt{{f}_{n{\bf{k}}}^{{\rm{DFT}}}({E}_{F}+\nu ,\sigma ){f}_{n{\bf{k}}}^{{\rm{Wan}}}({E}_{F}+\nu ,\sigma )}$ is an effective Fermi-Dirac distribution, and *f*(*E*_*F*_ + *ν*, *σ*) is the Fermi-Dirac distribution for DFT and Wannier interpolated states, with *E*_*F*_ being the Fermi level of the system. For insulators, any value of the Fermi level within the band gap is correct. Therefore, for insulators or semiconductors, we use the conduction band minimum (CBM) instead of *E*_*F*_, to ensure that we also assess the accuracy of the conduction bands in the first 2 eV above the CBM. In the following, we choose *ν* = 2 eV and *σ* = 0.1 eV (the same as in ref. ^21^) in order to consider band differences only for those bands with energy (approximately) below *E*_*F*_ + 2 eV. This includes the valence bands and a few conduction bands near the Fermi level, which are typically the relevant ones to determine most physical properties. Furthermore, in the calculations presented in this work, all computations based on PDWFs were configured with an additional frozen energy window, which was set to include all states up to 2 eV above the Fermi level for metals, or up to 2 eV above the CBM for insulators and semiconductors. This approach (also named PD+ED in ref. ^21^) is the recommended approach when using the PDWF method (see ref. ^21^) as it generally provides higher-quality Wannier interpolation than just using PD with no frozen energy window.

Identifying an appropriate disentanglement window facilitates Wannierization^17,21^. Given the vast number of material structures we address, a reasonable projectability window has been selected: bands are frozen when ${p}_{m{\boldsymbol{k}}} > {p}_{{\rm{thr}}}^{\max }=0.95$ and discarded when ${p}_{m{\boldsymbol{k}}} < {p}_{{\rm{thr}}}^{\min }=0.01$. The frozen energy window is set to 2 eV above *E*_*F*_ (or CBM) to reproduce the states around the Fermi energy or the band edges, while avoiding at the same time to include too many low-projectability high-energy bands. If convergence fails with these parameters, other values for ${p}_{{\rm{thr}}}^{\max }$ and ${p}_{{\rm{thr}}}^{\min }$ on a grid are attempted, as detailed in “Methods” section.

### Spin-orbit coupling

SOC, as a relativistic effect, plays a significant role in systems involving heavy elements. It can lift degeneracies^35^ and open band gaps at certain *k*-points where SOC plays a dominant role^36^, leading to notable changes in the electronic properties. Relativistic effects are described by the Dirac equation^37^, whose solutions are four-component spinors. However, two of these components correspond to antimatter, which is typically neglected in low-energy physics. As a result, SOC is often approximated as a relativistic correction to the Schrödinger equation^38^, with wavefunctions described as two-component spinors. In practice, fully relativistic (FR) pseudopotentials^39,40^ must be used to include SOC effects in DFT calculations (with “fully relativistic” we do not indicate solving the 4-component Dirac equation, but rather using pseudopotentials incorporating comprehensive relativistic effects, including SOC). Consequently, FR pseudopotentials are an essential ingredient for the computation of SOC-dependent properties^5,15^. The initial projectors for MLWFs are therefore PAOs obtained from these FR pseudopotentials. Since currently the SSSP^34^ library does not offer a SOC version, we performed our SOC calculations using the PseudoDojo^41^ and the modified-pslibrary^42^ sets, described in more details in the Methods section.

Due to SOC, the orbital quantum number *l* and the spin quantum number *s* are no longer good quantum numbers, and the system is instead described by the total angular momentum quantum number *j*. Consequently, the projectors become *j*-dependent when SOC is considered. To extend the workflow to SOC systems, we adjust the number of energy bands and projectors in the SOC system. For the PDWF approach, since the projectors can be directly obtained from the pseudopotential files, we modified the pw2wannier90.x code, part of QUANTUM ESPRESSO (QE)^43,44^, for projecting from plane-wave functions onto projectors accounting for their dependence on *j*. Additionally, to enable the functionality of reading projectors from external files as implemented in ref. ^21^, we also implemented routines for reading *j*-dependent projectors from external files.

**Fig. 1:**
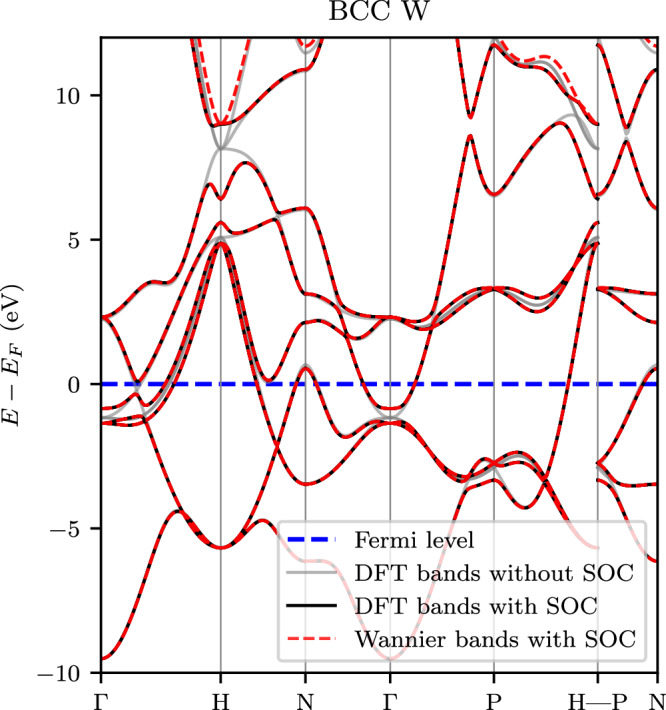
Electronic band structure of BCC tungsten. The gray and black solid lines are the energy bands obtained directly from first-principles DFT calculations without and with SOC, respectively. The red dashed lines are the energy bands obtained from Wannier interpolation with SOC using our extended PDWF method. The Fermi level is marked as a horizontal blue dashed line.

The effect of SOC on the band structure of BCC tungsten, as well as the quality of the Wannier-interpolated bands obtained with our algorithm including SOC, are demonstrated in Fig. 1. Some bands exhibit splitting due to SOC, particularly along the Γ − H *k*-path, where some band crossings transition into anti-crossings induced by SOC just below the Fermi level. The Wannier-interpolated band structure obtained using our extension of the PDWF method displays a *η*_2_ band distance of only 2.24 meV. Notably, even at approximately 8 eV above the Fermi level, the Wannier-interpolated bands remain in good agreement with the DFT bands. Thus, this Wannier tight-binding model is capable of accurately describing the electronic bands of tungsten and is suitable for precise calculations of SOC-related properties.

To further validate our algorithm, we performed Wannierization calculations including SOC on the set of 200 chemically diverse materials extracted from the Materials Cloud 3D crystals database (MC3D)^45^ already used for benchmarking in refs. ^20,46^. 173 among these materials contain elements with atomic numbers greater than 20, where SOC effects become non-negligible. Therefore, this dataset is a also good test set for estimating the accuracy of Wannier interpolation with SOC, and where the few materials containing only light elements enable us to verify that the robustness of the PDWF approach is not disrupted when including SOC effects, even when these are negligible.

**Fig. 2:**
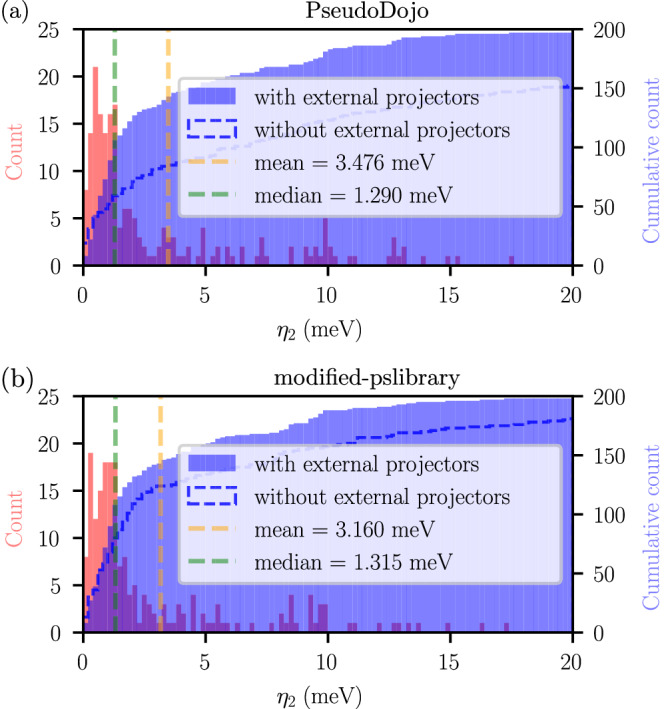
Band distance *η*_2_ of 200 systems with different pseudopotentials, including SOC effects. Histogram (red) and cumulative histogram (blue) of the band distance *η*_2_ of 200 spin-orbit coupling systems with different pseudopotentials sets: **a** the PseudoDojo library, and **b** the modified-pslibrary set (see Methods section). External hydrogenic atomic orbitals are introduced to the projectors to enhance the robustness of the PDWF method, making results obtained with the two pseudopotential libraries qualitatively very similar. As a comparison, the blue dashed lines are the cumulative histogram of *η*_2_ without introducing such external projectors, exhibiting a lower success rate and a strong dependence on the pseudopotential library. The orange (green) vertical line is the mean (median) band distance *η*_2_ of the 200 structures with external projectors; their values are shown in the legend of each panel. 98.5% (99%) of the 200 structures can be interpolated with a resulting *η*_2_≤20 meV when using PseudoDojo (modified-pslibrary), once hydrogenic atomic orbitals are introduced.

To compare the performance of different pseudopotential sets used to obtain PDWFs in the presence of SOC, we computed the band distance *η*_2_ using both the PseudoDojo and the modified-pslibrary sets described earlier. The cumulative histogram is shown with dashed lines in Fig. 2. When using PseudoDojo there are 48 cases with band distance *η*_2_ exceeding 20 meV, and the median and mean *η*_2_ are 6.243 meV and 20.772 meV, respectively. When using the modified-pslibrary set, there are instead only 19 cases with *η*_2_ larger than 20 meV, with median and mean *η*_2_ being 1.970 meV and 10.530 meV, respectively.

We note that even though both sets of calculations involve the same set of materials, the results exhibit significant differences. Moreover, we still have few materials with large *η*_2_ in both sets. We discuss how to address and solve both these issues (dependence on the pseudopotentials used, and low-quality band interpolation) in the next section, thanks to the inclusion of selected additional hydrogenic projectors. Nevertheless, we highlight that the quality of the results is already quite good, with the modified-pslibrary set (including SOC) achieving key metrics (median and mean band distance) comparable to the data from ref. ^21^ (PDWF without SOC), and the statistical results of the PseudoDojo still outperforming those obtained for HT calculations using the SCDM method^20,21^.

### Improving robustness by adding hydrogenic projectors

In this section, we discuss the motivation and effectiveness of extending the projection space by adding external projectors. We first observe that for several systems (11 out of 200), there remains a deviation between the Wannier interpolated bands and the DFT bands (*η*_2_ > 20 meV), with many exhibiting very large deviations (8 out of 200 systems have *η*_2_ > 40 meV). Similar results were obtained in the HT calculations in ref. ^21^ (478/21737 systems with *η*_2_ > 20 meV, where a less stringent criterion was used for insulators, using the value of *E*_*F*_ returned by the code rather than the CBM in the definition of *η*_2_). Analogously, our results using the PDWF method extended to SOC also exhibit similar trends, as discussed in the previous section. Furthermore, the statistical results of band distance depend on the pseudopotential sets: as discussed earlier, when using PseudoDojo, 48/200 (~24.0%) of the materials have *η*_2_ ≥ 20 meV, while 19/200 (~9.5%) have *η*_2_ ≥ 20 meV when using the modified-pslibrary set. Since physical quantities should not depend on the pseudopotential choice, and since the implementation of the calculation of certain properties may require specific pseudopotentials (e.g., advanced properties might be implemented only for norm-conserving pseudopotentials), it is important to devise algorithms that are largely independent of the underlying pseudopotentials. To address this, we aim to identify the causes of remaining discrepancies between DFT and Wannier-interpolated bands, and design an appropriate algorithm that can be easily applied across various pseudopotential sets, thereby enhancing the reliability and robustness of the PDWF method.

**Fig. 3:**
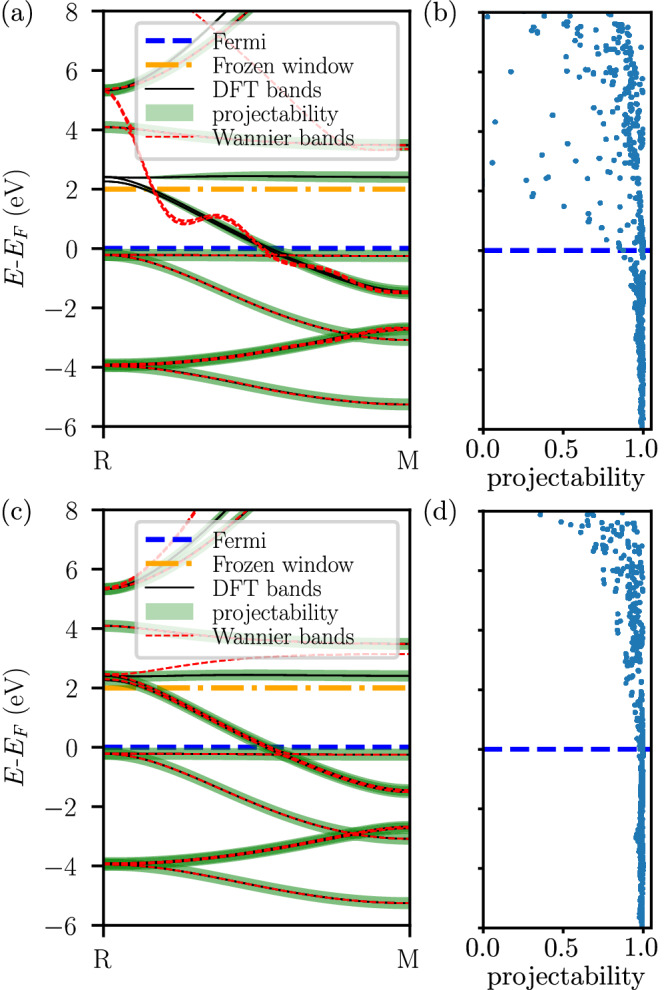
Effect of the introduction of additional hydrogenic projectors on the band structure and projectability of AlCo. **a** DFT bands (black solid lines) compared with Wannier-interpolated bands (red dashed lines) along the R−M path for AlCo without additional hydrogenic projectors, including only the orbitals from the pseudopotential files in the PseudoDojo library^41^: 3*s*, 3*p*, 3*d*, 4*s* orbitals for cobalt, and 3*s*, 3*p* orbitals for aluminum. The Fermi level and the top of the frozen window are marked with a blue dashed line and an orange dash-dotted line, respectively. The projectability (green fat bands) along *k*-points clearly illustrates the lack of projectability at the R point near the frozen window. **b** Projectability for all *k*-points for the system of panel (**a**). The projectability starts to decrease rapidly when the energy is larger than the Fermi level. **c** DFT bands compared with Wannier-interpolated bands when additional hydrogenic 4*p* orbitals are included for cobalt. **d** Projectability for all *k*-points for the system of panel (**c**). With the help of hydrogenic AOs, the projectability remains close to one up to a few eV above *E*_*F*_.

We selected AlCo as an example, for which we computed PDWFs using the PseudoDojo set, obtaining a fairly large *η*_2_ = 45.8 meV. The primary source of the difference between DFT and Wannier bands is along the *k*-path from R $\left(1/2,1/2,1/2\right)$ to M $\left(1/2,1/2,0\right)$, as shown in Fig. 3a. Notably, there are significant oscillations in the Wannier-interpolated bands near the Fermi level. To identify the source of the error, we examine the projectability of the Bloch states onto the trial PAOs within the first Brillouin zone in Fig. 3b. The data shows that the projectability remains close to 1 below the Fermi level, but gradually decreases at the Fermi level and above. Notably, already at 2 eV above the Fermi level there are states with essentially zero projectability (the states at the R point). Tracing the *k*-path from R to M, the projectability increases smoothly from 0, with approximately 0.3 projectability at the *k*-point $\left(1/2,1/2,1/3\right)$, which is the neighborhood of R on the *k*-path. However, since we employ the PD + ED algorithm, where ED freezes all bands below *E*_*F*_ + 2 eV for metals, the disentanglement process will keep these bands unchanged. Consequently, the almost-zero projectability at R leads to the exclusion of this band during the disentanglement, replacing it with a new band constructed from linear combinations of higher-energy bands. As a result, this forces a discontinuity (in reciprocal space) for this band, that is reflected in large oscillations of the resulting Wannier-interpolated bands.

Considering that we wish to maintain the accuracy of the interpolated bands within the *E*_*F*_ + 2 eV window, an approach to solve the issue described above for this system is to increase the projectability of bands near the energy window. Increased projectability can then only be achieved by extending the projection space. The most intuitive approach is thus to introduce external projectors to expand the Hilbert space of the projections, thereby enhancing the overall projectability. This was already considered in ref. ^21^, augmenting the number of projectors for silicon to include also *d* states.

There are several approaches possible to obtain external projectors. One approach consists in generating additional projectors using the pseudopotential generation code, and releasing new pseudopotentials. This approach is naturally the most physically accurate and can ensure maximal orthogonality between projectors, while providing the most precise projectability. However, it requires additional manual adaptation for different elements and pseudopotential libraries. Another approach expands the projectors by referencing other libraries, such as complementing missing projectors between pslibrary and PseudoDojo, or directly obtaining projectors from third-party libraries, like extracting the desired projectors from the PAOs obtained from the OpenMX code^21,47^. However, these often yield projectors defined on different radial coordinates, requiring separate projection calculations or interpolation to align the projectors on a common coordinate system. Moreover, this approach also introduces a higher complexity, depending on several external libraries. Finally, there is no guarantee that the projectors obtained from different libraries are orthogonal to each other. The approach that we will adopt in the following relies instead on adding hydrogenic atomic orbitals. Standard hydrogenic-orbital projectors have been widely used in past applications, and have been effectively applied in HT calculations^19^. Furthermore, since a hydrogenic AO is written using analytical expressions, it can be easily evaluated on the radial coordinates of the original projectors.

As a supplement to the standard hydrogenic approach, we have extended the radial function expressions to accommodate different angular quantum numbers (since the radial part in general depends also on the angular quantum number *l*, in addition to the principal quantum number *n*). The various radial functions listed in Table 1 can cover all the projectors needed for common elements. The shape of these radial functions is controlled by the parameter *α*. Therefore, in order to define which hydrogenic AOs to use to expand missing projectors, we only need a table containing the minimal required orbitals for each chemical element, together with their corresponding *α* values. However, compared to projectors generated directly using pseudopotential generation codes, using hydrogenic AO projectors inevitably introduces larger overlaps between AOs and the pseudopotential PAOs, which impacts projectability. While intuitively this is not expected to be an issue for the added projectors, since these are are typically needed only to complete the Hilbert space for describing higher-energy bands, we conduct tests to evaluate the effectiveness of this algorithm.

**Table 1:** Analytical expressions for the radial part of hydrogenic AOs with different number of radial nodes (*n*_*r*_ = *n* − *l* − 1, where *n* is the principal quantum number) and angular quantum numbers *l*

number of nodes (*n*_*r*_)	0	1	2
*s* (*l* = 0)	$2{\alpha }^{3/2}\exp (-\alpha r)$	$\frac{1}{2\sqrt{2}}{\alpha }^{3/2}(2-\alpha r)\exp (-\alpha r/2)$	…
*p* (*l* = 1)	$\frac{1}{2\sqrt{6}}{\alpha }^{3/2}\alpha r\exp (-\alpha r/2)$	$\frac{4}{81\sqrt{6}}{\alpha }^{3/2}(6\alpha r-{\alpha }^{2}{r}^{2})\exp (-\alpha r/3)$	…
*d* (*l* = 2)	$\frac{4}{81\sqrt{30}}{\alpha }^{3/2}{\alpha }^{2}{r}^{2}\exp (-\alpha r/3)$	…	…

Only the expressions for the values of *n*_*r*_ and *l* that are needed for covering the missing orbitals in the periodic table are reported.

When considering the minimum required orbitals, we follow the principle of including one additional higher AO for elements within the same period. For elements in the *N*-th period, if *N* = 1, only the 1*s* orbital is considered. For *N* = 2 or 3, both *Ns* and *Np* orbitals are added to the requirements list. For elements in higher periods, alkali and alkaline earth metals require *Ns* and (*N* − 1)*d* orbitals, transition metals need *Ns*, *Np* and (*N* − 1)*d* orbitals, and elements from the boron group to the noble gases require *Ns* and *Np*. According to these rules, we can list the minimal set of required orbitals for each element, which is also provided explicitly in the Supplementary Table II. Note that some of these orbitals might be redundant (i.e., having almost zero projectability on states below the frozen energy window). However, with the aim of maximizing the success rate for a fully automated algorithm, we include all of them. An analysis of the projectability of the individual orbitals could be performed as a post-processing step to determine which can be removed, if a minimal Wannier basis set is desired.

We stress that any additional hydrogenic projector should be orthogonal to the existing PAOs from the pseudopotential. In particular, if the pseudopotential PAOs already include an orbital with the same angular quantum number *l* but smaller principal quantum number *n*, the additional projector should use a radial function that contains a node. To obtain a table of appropriate *α* values, we employed two approaches. For projectors with a node in the radial function, we adjust *α* to ensure that the inner (PAOs) and outer (added hydrogenic AO for which we need to determine *α*) projectors are orthogonal. For projectors without nodes (i.e., when the pseudopotential does not already include an inner-shell projector with the same *l*), instead, we derive the *α* value through fitting of our analytical expressions (Table 1) to the PAOs from the OpenMX code^47^. Because of the orthogonality condition to the underlying PAOs, the values of *α* will depend on the chosen pseudopotential library. The complete list of projectors corresponding to each pseudopotential library used in this work, including (i) supplementary hydrogenic AOs with their associated *α* values and sources, and (ii) PAOs obtained directly from pseudopotentials, can be found in Supplementary Tables III, IV, V, and VII. In practice, in most cases we use PAOs from the pseudopotentials: e.g., 79.88% (87.17%) of the starting projections when using the PseudoDojo (modified-pslibrary), see Supplementary Tables V and VII. We extend with hydrogenic AOs with α parameter set by orthogonalizing them to underlying PAOs with same orbital character in 11.45% (7.07%) of the cases. Finally, we resort to hydrogenic AOs with α parameter obtained by fitting the PAOs from OpenMX in the remaining 8.67% (5.76%) of the cases.

Despite these precautions, the added orbitals are in general not orthogonal to the PAOs. Therefore, we apply a Gram–Schmidt orthonormalization^48^ when external projectors are added. More precisely, we first perform separately two Löwdin orthonormalizations for the PAOs from the pseudopotentials and for the external hydrogenic projectors. Then, we fix the PAOs projectors and perform a further Gram–Schmidt orthonormalization step on the hydrogenic orbitals only, ensuring that the full set (PAOs + additional hydrogenic orbitals) form an orthonormal set. This procedure allows us to faithfully keep the PAOs from pseudopotentials unchanged, while at the same time making sure that external projectors are always orthonormal to pseudopotential PAOs. Instead, a single Löwdin orthogonalization of all orbitals would distort the PAOs, an undesirable effect since they are accurately describing the system chemistry. For a detailed description of the orthonormalization procedure, see Supplementary Section V.

The algorithms described above have been implemented and will become available in the next releases of QE^43,44^ (pw2wannier90.x code). In addition, we can use the scripts in the AiiDA-Wannier90-Workflows repository^49^ (folder dev/projectors) to extract PAO information from the pseudopotentials and determine the minimal required additional projectors and the corresponding *α* values, according to the method described above. The script then generates the missing projectors and exports them as a .dat file, which serves as an additional input for pw2wannier90.x.

Going back to the example of Fig. 3, comparing the PAOs in the PseudoDojo with the minimum required AOs, we hypothesized that adding an additional 4*p* AO for cobalt could enhance the overall projectability. The computational results support this hypothesis: after introducing a 4*p* external projector, the minimum projectability near the upper limit of the frozen energy window increased to approximately 0.8, see Fig. 3d, which is sufficient to maintain continuity between adjacent *k*-points using the PD+ED disentanglement approach. The spread of the cobalt WFs decreased from a range of 0.73–2.46 Å^2^ to 0.46–1.14 Å^2^, confirming an increased smoothness of the wavefunctions in reciprocal space, thus resulting in more localized WFs and in highly accurate Wannier-interpolated bands (up to 2 eV above *E*_*F*_), with *η*_2_ = 2.34 meV, see Fig. 3c.

The computational results for the 200 materials with SOC, before and after introducing the external projectors, are shown in Fig. 2. The statistical data shows significant improvements. After adding external hydrogenic projectors to PAOs from PseudoDojo (modified-pslibrary), the mean *η*_2_ decreases from 20.772 (10.530) meV to 3.476 (3.176) meV, and the median *η*_2_ drops from 6.243 (1.970) meV to 1.290 (1.315) meV. Notably, the computational success rate increases from 76.0% (90.5%) to 98.5% (99.0%) when the PseudoDojo (modified-pslibrary) is used, with the largest *η*_2_ among all systems being just above 20 meV: 25.30 (25.37) meV, respectively. The ${\eta }_{0}^{max}$ also shows a significant reduction as shown in Supplementary Fig. 3a–d, with the mean ${\eta }_{0}^{max}$ value decreasing from 165.255 (66.474) meV to 8.387 (7.803) meV and the median ${\eta }_{0}^{max}$ also decreasing from 13.204 (7.739) meV to 6.223 (5.784) meV. Notably, the maximum value of ${\eta }_{0}^{max}$ decreases from 2033.1 (1880.6) meV to 39.50 (42.78) meV. This clearly illustrates that our approach improves the accuracy of Wannier interpolated bands within the valence bands, even when using the stringent measure ${\eta }_{0}^{max}$. Therefore, by appropriately expanding the projection space, we both improve the robustness of the PDWF approach and strongly mitigate the pseudopotential dependence of the results.

Introducing additional hydrogenic projectors increases the matrix sizes, thus one might think that it would also increase the computational cost. However, the higher success rate actually allows the Wannierization process to converge in fewer iterations and reduces the need of resorting to the optimization of the PDWF parameters ${p}_{{\rm{thr}}}^{\max }$ and ${p}_{{\rm{thr}}}^{\min }$, as discussed in the Methods section, thus significantly reducing the overall computational time. E.g., for the systems using pseudoDojo (modified-pslibrary), after incorporating hydrogenic projectors the number of systems needing PDWF parameter optimization decreases from 65 (31) to 14 (12), and the total number of optimization iterations reduces from 1499 (682) to 123 (81), resulting in a reduction from 14,200 (9100) to 6300 (5400) CPU core hours on the hardware used for these simulations. We also note that, after introducing hydrogenic projectors, Fig. 2 reveals that the band distance counterintuitively slightly increases in the region of very low *η*. We analyzed these data points in detail in Supplementary Section III, finding that even when band distance increases, it remains below 4 meV for most materials. Hence, this effect remains negligible and well within our thresholds for successful interpolation.

### Extended validation in spin-unpolarized systems

**Fig. 4:**
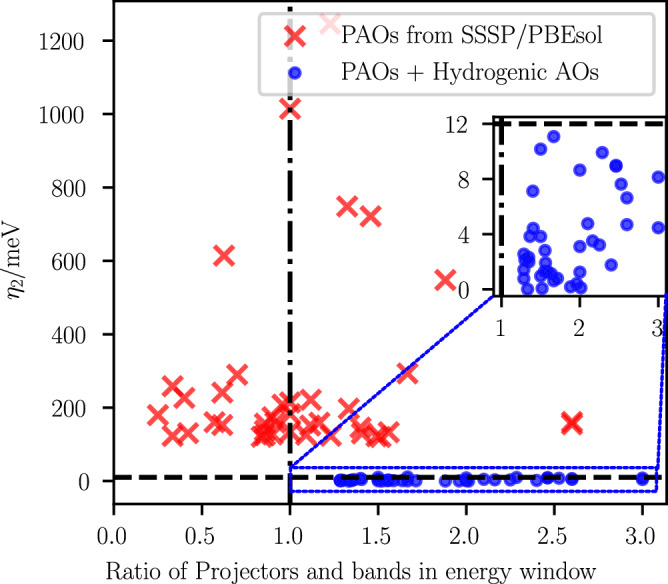
Results of recalculating the 40 systems with largest band distance from ref. ^21^. The red crosses are the original data directly from ref. ^21^, whose projectors are PAOs from pseudopotentials in SSSP PBEsol Efficiency v1.1. The blue circles are the recalculated data where the same PAOs are complemented by additional hydrogenic AOs. The blue dashed rectangle area (which includes all blue circles) is zoomed in the inset, showing that after adding hydrogenic AOs, all 40 materials have a *η*_2_≤12 meV, thus demonstrating the effectiveness of our algorithm even in extremely challenging cases.

In addition to the results on SOC systems mentioned above, we also conducted tests of our extended PDWF method with added hydrogenic projectors on non-SOC systems. To stress test our approach, we first consider in Fig. 4 the 40 systems that resulted in the largest band distance among the 21,737 HT results of ref. ^21^, confirming that the introduction of external projectors allows us to achieve convergence for all of them. Each point in Fig. 4 represents a structure, where the *x* coordinate is the ratio between the number of projectors (i.e., of Wannier functions) and the number of bands within the *E*_*F*_ + 2 eV (or CBM + 2 eV) window, while the *y* coordinate is *η*_2_. (Note that the energy window of *E*_*F*_ + 2 eV or CBM + 2 eV is not exactly the frozen window, but it is instead the cut-off window for calculating the average band distance *η*_2_. Moreover, for materials having more bands than projectors in the energy window, the workflow automatically lowers the value of the top of the frozen window to avoid crash in Wannierization and attempt to still achieve a Wannierization). Since each projector can correspond to only one band state, and considering that disentanglement involves a linear combination of several bands, when the number of projectors is less than (or just slightly more than) the number of bands within the energy window, the disentanglement process does not have enough information from the initial projections, possibly resulting in a poor Wannier interpolation. These results clearly indicate that the inaccuracy of the Wannier interpolated bands for many of these materials was due to the lack of a sufficient number of projectors. After adding external projectors (and enabling the guiding center setting during the Wannierization process), the *η*_2_ for all systems was reduced to below 12 meV (blue circles in Fig. 4). This demonstrates that the additional hydrogenic projectors can significantly enhance the robustness of the Wannier interpolation also in non-SOC systems, even in extremely challenging cases.

**Fig. 5:**
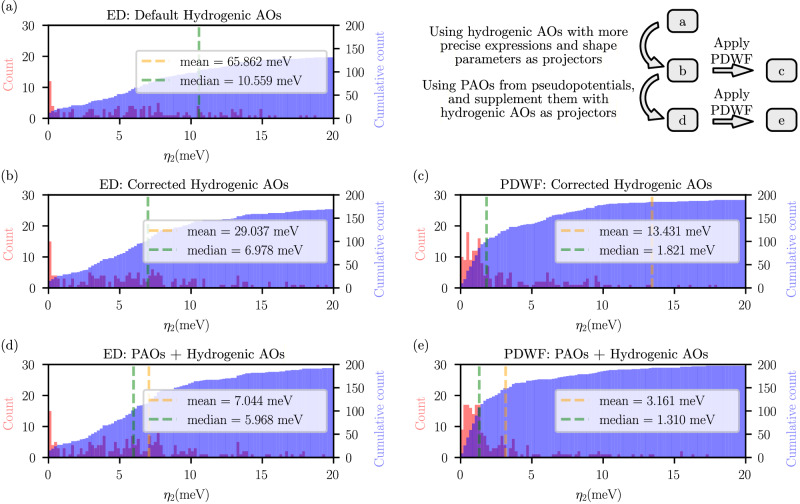
Summary of the effect of several ingredients of the extended PDWF algorithm on the quality of Wannier interpolation. Histogram (red) and cumulative histogram (blue) of band distance *η*_2_ for our test set of 200 structures, using different algorithms and projectors (all without SOC). All sets of calculations use the same number of projectors and same configurations of semi-core states. All frozen windows were set to *E*_*F*_ + 2 eV (CBM + 2 eV) for metals (insulators and semiconductors). **a** ED with the default all-hydrogenic AOs; **b** ED with the corrected all-hydrogenic AOs (see Table 1); **c** PDWF with the corrected all-hydrogenic AOs; **d** ED with PAOs and external hydrogenic AOs; and **e** PDWF with PAOs and external hydrogenic AOs. The orange (green) vertical lines are the mean (median) band distance *η*_2_, whose values are shown in the legend of each panel. The difference and the relation between each set of data are shown in the top right panel. Additional comparisons with further combinations of disentanglement method and starting projectors can be found in Supplementary Section IV.

We also consider the same set of 200 systems using for validating the extension to SOC, see Fig. 5e. Also in this case, our approach generally reduces the *η*_2_ values, with the mean *η*_2_ decreasing from 7.625 meV to 3.169 meV and the median *η*_2_ dropping from 2.690 meV to 1.310 meV with respect to the results of ref. ^21^. (Note that since we consider here the band distance up to CBM + 2 eV for systems with band gap, rather than up to *E*_*F*_ + 2 eV as in ref. ^21^, the values reported here slightly differ quantitatively from those reported in ref. ^21^.) Furthermore, similarly to the SOC case, we achieve a 98.5% success rate with the criterion of *η*_2_ ≤ 20 meV. As shown in Supplementary Fig. 3, mean and median ${\eta }_{0}^{max}$ also show significant reduction from 32.898 meV to 8.858 meV, and from 8.658 meV to 6.276 meV, respectively. The maximal ${\eta }_{0}^{max}$ reduced from 901.1 meV to 41.99 meV.

To further illustrate how the various components of the extended PDWF method jointly contribute to the overall final robustness of the algorithm, we compare in Fig. 5 the results obtained on the 200-structure set with different projection methods. SOC is not included in these calculations, and the SSSP PBE Efficiency v1.1 pseudopotential library is used, to enable direct comparison with the results of ref. ^21^. Panel (a) of Fig. 5 shows the results of the standard Souza–Marzari–Vanderbilt ED algorithm, using as starting projections the common analytical hydrogenic AOs (as, e.g., defined in the Wannier90 code^50,51^). The pseudopotential PAOs are used in this case solely to determine the angular character (*s*, *p*, *d*, …) of the projectors to consider, but no further information is extracted from the pseudopotential projectors. Furthermore, the default values for *α* and for the projector analytical shapes are employed, as defined in the Wannier90 code. While the approach, that has been widely used in the literature, is able to provide a good Wannier interpolation for over 50% of the systems, it still exhibits a significant number of failures, with a mean *η*_2_ of 65.862 meV and a median *η*_2_ of 10.559 meV.

As discussed earlier, however, the radial part of the hydrogenic orbitals should depend also on the angular quantum number *l*, meaning that different expressions must be applied for radial functions with *s*, *p*, or *d* angular character (see Table 1). Using these corrected radial functions as projectors, combined with optimized values of *α* (see Supplementary Table IV) obtained by fitting the corresponding PAOs radial functions (and complementing with fitting from OpenMX where PAOs are missing), results in panel (b) of Fig. 5. The success rate significantly increases, and the mean (median) *η*_2_ decreases to 29.037 (6.978) meV, demonstrating that an improved choice of radial functions and *α* values already enhances the quality of the Wannier interpolation.

Starting from panel (b), two possible directions can be taken to further improve the quality of the Wannier interpolation. The first is to use PDWFs instead of the ED algorithm, see panel (c) of Fig. 5. The mean (median) *η*_2_ further reduces to 13.431 (1.821) meV, demonstrating that the PDWF algorithm can provide a more accurate Wannier interpolation than the standard ED algorithm, even when using the same projectors. (As a note, the large mean *η*_2_ values in panels (a-c) are due to few cases with very large *η*_2_ that are not visible in the histogram as they are outside of the *x*-axis range.) Alternatively, one can add external hydrogenic AOs to the PAOs, as shown in panel (d) of Fig. 5. The mean (median) *η*_2_ also reduces, with respect to panel (b), to 7.044 (5.968) meV, demonstrating that the addition of external hydrogenic AOs also plays an important role in improving the Wannierization robustness.

Finally, panel (e) of Fig. 5 represents our improved algorithm, combining PDWF and the addition of hydrogenic AOs. The resulting Wannier interpolated bands exhibit the highest quality, with the lowest values for the mean (median) *η*_2_ of 3.161 (1.310) meV, and such a combination achieves a 98.5% success rate, defined as systems having *η*_2_ ≤ 20 meV.

These results demonstrate that, although hydrogenic AOs are not as physically precise as the projectors from the pseudopotentials, they are still essential as a complement to PAOs when these are not sufficient to cover all states in the frozen window. The additional advantage is that the present combined method essentially eliminates the dependence of PDWFs on pseudopotentials. In fact, while different pseudopotentials may have different sets of PAOs, discrepancies are minimized by introducing hydrogenic functions to complete the projectors to the same list of orbitals (for a given chemical element), see Supplementary Table II. We also stress that good interpolation quality can only be achieved if enough PAOs are already included into the pseudopotential (as these capture the detailed chemistry of the material close to the atoms), and we need to possibly add only a few more hydrogenic AOs to recover a large enough projectability for the high-energy bands in the relevant energy range. Indeed, if no PAOs are available at all in the pseudopotentials, only considering hydrogenic AOs is equivalent to panel (b) of Fig. 5, showing that a much lower interpolation quality would be achieved.

The present method thus offers a flexible and straightforward approach to generate the needed additional projectors without requiring to execute and possibly modify the pseudopotential generation codes, making it a practical algorithm for enhancing the performance of PDWFs for high-throughput research.

### Magnetization

We finally discuss the extension of the PDWF workflows to magnetic systems. In magnetic DFT calculations, one typically distinguishes between a collinear and non-collinear magnetic treatment. The former indicates that the spin can only be polarized along a given quantization axis (e.g., *z*), which is often appropriate to describe ferromagnetic and collinear antiferromagnetic materials, but can fail to accurately describe the magnetic structure of more complex systems where magnetic moments are not simply parallel or antiparallel, such as non-collinear antiferromagnetic systems or spin spiral states. In a collinear treatment, spinor wavefunctions have one of the two components being identically zero. In QE, the code therefore considers collinear calculations by only storing the non-zero component of the spinor wavefunctions, effectively treating each of the two spin channels as spin-unpolarized calculations, but with a doubled set of *k* -points (one set for spin up, one for spin down). (Note that this is possible only in the absence of SOC, so that the Hamiltonian does not mix the two spin channels). Consequently, in our workflow design, we choose to separate the spin-up and spin-down calculations, and later merge the band structures after the calculations are completed.

Instead, since formally the treatment of non-collinear spin systems is the same as that of SOC systems, because also in this case the wavefunctions are two-component spinors, the corresponding workflow structure is analogous to that of SOC calculations, with the only additional requirement being a tool to incorporate the magnetic moment as an input. Within the AiiDA-Wannier90-Workflows repository^49^ we have developed a new MagneticStructureData data plugin for AiiDA (available in the aiida_wannier90_workflows.data.structure Python package) that processes magnetic moment structures and organizes input files. In the following we briefly discuss the results of the verification of our workflows for collinear and non-collinear magnetic systems.

**Fig. 6:**
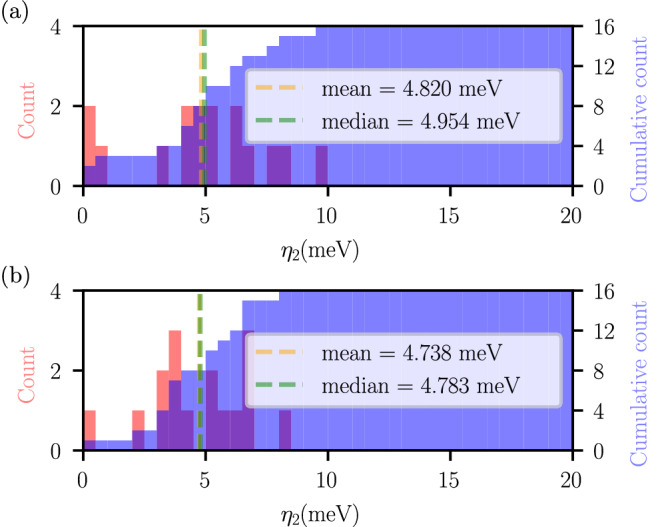
Results of the extended PDWF workflows for magnetic (collinear and non-collinear) systems. Histogram (red) and cumulative histogram (blue) of the band distance *η*_2_ of (**a**) 16 collinear magnetic systems and **b** 16 non-collinear magnetic systems. The orange (green) vertical lines are the mean (median) band distance *η*_2_, whose values are shown in the legend of each panel. In both cases, SSSP pseudopotentials and additional external hydrogenic projectors were used to obtain PDWFs.

We refer to collinear magnetic systems as those simulations where spin-polarized calculations were performed without activating the spin-directional degree of freedom. We performed calculations on 16 relevant magnetic systems, including collinear ferromagnetic body-centered cubic (BCC) iron, collinear antiferromagnetic Mn_2_F_4_, and non-collinear antiferromagnetic *L*1_2_ IrMn_3_, as well as other magnetic systems primarily composed of Fe, Co, Ni, and Mn elements. Note that for magnetic materials with non-collinear configurations (e.g., IrMn_3_), we nevertheless restrict them in this section to collinear magnetic configurations for testing purposes. In this set of calculations, the initial magnetic moments in DFT calculations are chosen to have the same magnitude as the *z* component of the actual 3D magnetic structure. Without applying further constraints, we allowed the DFT code to converge to the ground-state magnetic moment structure within the framework of a collinear ferromagnetic system. Our extended PDWF approach was used to accurately obtain the Wannier functions, incorporating external hydrogenic atomic orbitals into the projectors to enhance robustness, as detailed earlier. The calculated band distances are shown in Fig. 6a. Results on these systems exhibit mean and median *η*_2_ below 5 meV.

We then consider calculations using a non-collinear magnetization approach, i.e., where the spin degree of freedom is fully activated. This approach allows for accurate reconstruction of magnetic structures both for materials with parallel or antiparallel collinear magnetic configurations, as well as for truly non-collinear materials. Given the complexity of magnetic structures, also in this case we selected only 16 magnetic systems, including both collinear and non-collinear configurations, as well as ferromagnetic and antiferromagnetic systems. The detailed list of materials, with their magnetic configurations, is provided in Supplementary Section VI. Using the PDWF algorithm and external hydrogenic AOs, the calculated band distances are shown in Fig. 6b. Also in this case, the MLWFs generated by our extended PDWF method demonstrate an excellent performance in accurately describing electronic structures, with both mean and median *η*_2_ below 5 meV, thus showcasing the effectiveness of our extended PDWF algorithm in treating magnetic systems.

## Discussion

We present the extension of the PDWF algorithm to collinear and non-collinear magnetic systems, and to systems including SOC. Moreover, we further improve the robustness of the algorithm by defining an automated strategy for incorporating additional external (hydrogenic) projectors when the pseudopotentials do not include sufficient projectors to describe the lowest-energy unoccupied states. Our extended algorithms are fully automated using the AiiDA workflow engine^52–54^ and we make our implementation available open-source in the AiiDA-Wannier90-Workflows package^49^.

To benchmark the algorithms, we calculate PDWFs for multiple systems (spin-unpolarized, magnetic, and SOC systems) and demonstrate that the present extended PDWF algorithm can reliably and automatically construct MLWF-based tight-binding models for both magnetic and SOC systems. We find that essentially all deviations between Wannier-interpolated bands and DFT bands in earlier (spin-unpolarized) PDWF results^21^ can be attributed to the absence of all desirable projectors in the pseudopotentials. This can lead to poor projectability of important low-energy empty states, resulting in less localization of the corresponding Wannier functions and, in turn, in poorer interpolation of the electronic bands. The addition of external hydrogenic projectors can improve significantly the accuracy of PDWFs and minimize the dependence of Wannier interpolation results on the specific set of PAOs defined in the chosen pseudopotentials, achieving in our test set a success rate of over 98% (defined as an average band distance *η*_2_ ≤ 20 meV) when interpolating all bands up to the Fermi level + 2 eV for metals, and up to the CBM + 2 eV for insulators and semiconductors. Notably, if we consider *η*_1_ as a metric (bands up to 1 eV above the Fermi level or the CBM), all systems achieve *η*_1_ ≤ 15 meV (see Supplementary Information Sec. II A).

Finally, we compare the results of the Wannierization procedure obtained with various types of projectors (only hydrogenic AOs, PAOs, PAOs + external hydrogenic AOs) and we elucidate the key contributions in the extended PDWF algorithm to the overall quality of the Wannier interpolation. The present results underscore how PDWFs can be obtained in a systematic, robust and automated approach, enabling their straightforward and efficient application to several applications ranging from advanced property calculations^10,55^ to high-throughput materials discovery projects^56^.

## Methods

### Code implementation

We implemented projections with PAOs and hydrogenic AOs in the pw2wannier90.x executable, part of the QUANTUM ESPRESSO (QE)^43,44^ package since version 7.5. It can read user-provided custom projector files and calculate the projections of plane wave functions onto the projectors. The implementation supports SOC systems, non-collinear and collinear magnetic systems, as well as spin-unpolarized systems. The projector files can be generated using a script in the AiiDA-Wannier90-Worflows package^49^ based on the rules defined in Sec. II B. Moreover, we implemented a strategy to perform orthogonalization of the projectors by first applying Löwdin orthogonalization^18^ separately to the PAOs and to the hydrogenic AOs, and then fixing the PAOs (ensuring that they remain intact) and performing a subsequent Gram–Schmidt orthogonalization^48^ of the entire set of projectors, as discussed in the main text and in the Supplementary Information.

### DFT calculations

The DFT calculations are carried out using QE, with various pseudopotential libraries for different systems, as discussed in the main text. Specifically, and unless stated otherwise, for spin-unpolarized systems without SOC we use SSSP PBE Efficiency v1.1^34^. For SOC systems, we use the PseudoDojo PBE v1.4 (norm-conserving, FR) library^41^ and the pslibrary^42,57^ (PAW, FR) for comparison and validation. The HT calculations are managed with the AiiDA infrastructure^52–54^, which submits QE and Wannier90^51^ calculations to remote clusters, parses and stores the results into a database, while also orchestrating all sequences of simulations and workflows. The automated AiiDA workflows are open-source and hosted on GitHub^49^. To attach magnetic information to the crystal structure, we extend the AiiDA StructureData plugin to a new MagneticStructureData class, defined as part of the AiiDA-Wannier90-Workflows package. In the future, we plan to replace this custom class with the new StructureData class defined in the AiiDA-Atomistic^58^ plugin.

### Psuedopotential libraries in SOC calculations

In calculations including SOC, we use pseudopotentials from the PseudoDojo 0.4^41^ library containing norm-conserving FR pseudopotentials, and conduct validation calculations using the pslibrary^42^ 1.0.0 (PBE, PAW, FR). For pslibrary, we selected the corresponding pseudopotentials based on the recommendations from ref. ^57^. We note that, for the pslibrary, some pseudopotentials for certain elements may either be missing or result in DFT calculations that fail to converge. Consequently, for some elements, we used older versions of pslibrary or substituted pseudopotentials from PseudoDojo. Therefore, we refer to this set as a modified-pslibrary set in the main text; the specific pseudopotential files used are detailed in Supplementary Table VI.

### PDWF parameters optimization

We perform an automated optimization of the PDWF parameters in our workflows. We start by freezing states whose projectability is greater than ${p}_{{\rm{thr}}}^{\max }$ (default 0.95), and ignore states with projectability lower than ${p}_{{\rm{thr}}}^{\min }$ (default 0.01). If the current setting has more projectors than states in the disentanglement space, the workflow automatically reduces ${p}_{{\rm{thr}}}^{\min }$ using the values [0.005, 0.0025, 0.000125, 0]. If the average band distance *η*_2_ is below a threshold (set to 10 meV) after Wannierization, the workflow stops; otherwise the workflow will seek for another set of ${p}_{{\rm{thr}}}^{\max }$ and ${p}_{{\rm{thr}}}^{\min }$ that achieves this condition, following the strategy from ref. ^21^. In particular, when not adding hydrogenic projectors, ${p}_{{\rm{thr}}}^{\max }$ is iterated between 0.99 and 0.85 with a step size of 0.01, and ${p}_{{\rm{thr}}}^{\min }$ between the two values 0.02 and 0.01. When using PAOs and external hydrogenic projectors, instead, ${p}_{{\rm{thr}}}^{\max }$ is iterated on the values [0.99, 0.95, 0.90, 0.85], and ${p}_{{\rm{thr}}}^{\min }$ in [0.02, 0.01, 0.005, 0.0025, 0.00]. If *η*_2_ is still larger than the threshold after looping over all parameter combinations, the workflow outputs as optimized MLWFs the results of the Wannierization which resulted in the smallest *η*_2_ value. (Note that currently the *η*_2_ computed by the workflow is still defined as *E*_*F*_ + 2 eV rather than CBM + 2 eV, even in the cases of gapped systems. However, this is only the stopping condition of the workflow, while we stress that the discussion of *η* for insulators and semiconductors in the main text is based on the definition with CBM for gapped systems).

## Supplementary information

Supplementary Information

## Data Availability

All data generated in this work, as well as scripts to generate relevant plots, are available on the Materials Cloud Archive^59^ at 10.24435/materialscloud:9g-ds^60^. This entry also includes AiiDA^53^ archive files with the full provenance of all simulations and data.
