# Supplementary material for: Robust Wannierization including magnetization and spin-orbit coupling via projectability disentanglement
Source: NPJ Comput Mater. 2025 Nov 21;11(1):353. doi: 10.1038/s41524-025-01835-8 (PMC12638246; doi:10.1038/s41524-025-01835-8)
Supplement: Supplementary file 1 — Supplementary Information [file 41524_2025_1835_MOESM1_ESM.pdf]

Supplementary Material for: Robust Wannierization including magnetization and spin-orbit coupling via projectability disentanglement

Yuhao Jiang, Junfeng Qiao, Nataliya Paulish, Weisheng Zhao, Nicola Marzari, and Giovanni Pizzi

CONTENTS

I. SOC-weighted band distance 2

II. Band distance comparision 3
A. Band distance with lower energy window 3
B. Maximum band distance comparision 4

III. Band distance after introducing external projectors 5

IV. Additional comparisons between approaches for disentanglement and initial projections 5

V. Orthonormalization strategy 6

VI. Magnetic structures 8

VII. Required projectors set for a robust PDWF Wannierization 9

VIII. Detailed information on the projectors used in this work 10

References 26

I. SOC-WEIGHTED BAND DISTANCE

When studying SOC systems, it is expected that the Wannier functions can accurately reproduce the plane wave functions as closely as possible within the SOC-strongly-influenced k-space. In terms of band distance, the accuracy near the SOC-induced band splitting should be maintained at a level comparable to other regions. As shown in an artificial schematics in Supplementary Fig. 1(a), an oscillating interpolated band may have a similar average band distance to that of an interpolated band with a small rigid shift of all bands. However, it is evident that the oscillating energy band corresponds to Wannier functions that are insufficient to accurately describe the system, because of the mismatch of the band splitting and of the band derivatives (which are an important ingredient, e.g., of transport calculations). Therefore, we designed a SOC-weighted band distance metric to emphasize the accuracy of the Wannier-interpolated bands near the SOC-induced splitting.

Similar to the definition of the band distance, we define the SOC-weighted band distance as

$$\eta_{\nu}^{\mathrm{SOC}} = \sqrt{\frac{\sum_{n\mathbf{k}} \tilde{f}_{n\mathbf{k}}^{\mathrm{SOC}} \left(\epsilon_{n\mathbf{k}}^{DFT} - \epsilon_{n\mathbf{k}}^{Wan}\right)^2}{\sum_{n\mathbf{k}} \tilde{f}_{n\mathbf{k}}^{\mathrm{SOC}}}}, \qquad (1)$$

where $\tilde{f}_{n\mathbf{k}}^{\mathrm{SOC}} = \sqrt{\tilde{f}_{n\mathbf{k}} |\tanh\left(\left(\epsilon_{n\mathbf{k}}^{\mathrm{SOC}} - \epsilon_{n\mathbf{k}}^{noSOC}\right)/\sigma^{\mathrm{SOC}}\right)|}$, $\tilde{f}_{n\mathbf{k}}$ being the effective Fermi-Dirac distribution. The tanh function is used to provide a weight to each band and $k-$point contribution, dependent on the splitting between the corresponding bands with and without SOC. We selected a σ value of 50 meV, so that for instance a specific state with SOC splitting of 100 meV corresponds to a weight of 0.964, close to 1. In contrast, states without splitting or with very small splitting will have almost no weight.

In Supplementary Fig. 1(d), we can observe that most of the SOC-weighted band distance and the band distance are similar, proving that with our method the interpolation errors when SOC is included are not originating only SOC-specific errors, i.e., from regions where SOC has a large effect, but they are interpolation errors distributed across the BZ. The "standard" band distance can thus be safely used to describe the discrepancy between the Wannier-interpolated bands and the DFT bands also when SOC is included.

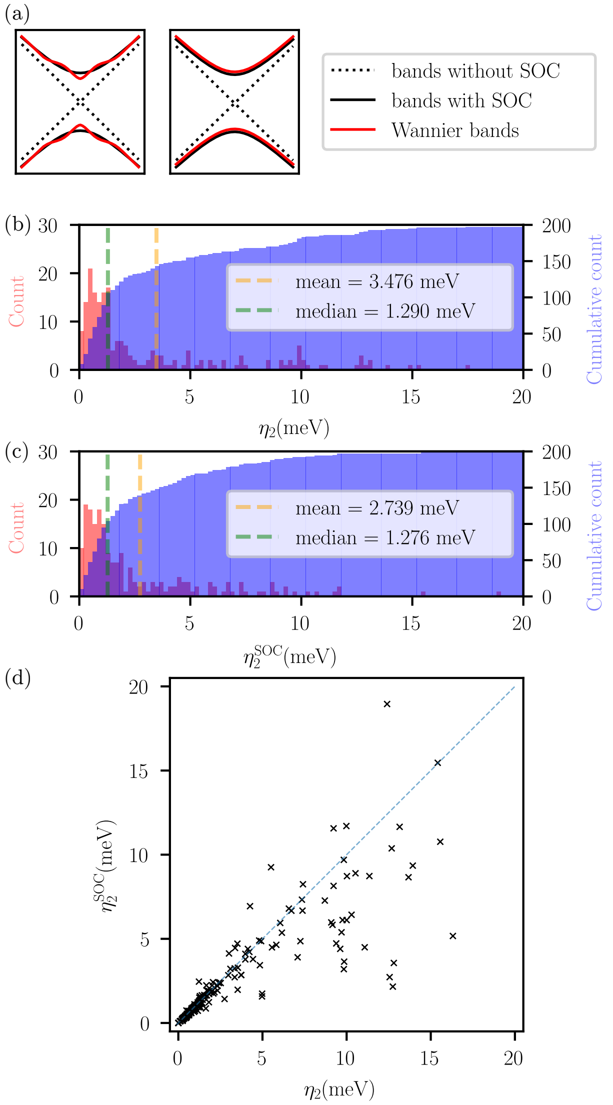

SUPPLEMENTARY FIG. 1. (a). Two sketches of Wannier interpolation of bands near a SOC-induced splitting. Note that the two sketches are only schematic representations and do *not* correspond to the band structure of any real system. In particular, we emphasize that the band discrepancies in the right panel would only occur for those k-points that were not included in the original DFT k-mesh. While the average band distance in the two sketches is approximately similar, the left figure exhibits significant oscillations near the SOC splitting, which is detrimental for accurately predicting properties dominated by SOC. Histogram (red) and cumulative histogram (blue) of (b) the band distance η_2 and (c) the SOC-weighted band distance η_2^{SOC}. The orange (green) vertical line is the mean (median) band distance, whose value(s) are shown in the legend of each panel. `PseudoDojo` 0.4 and external hydrogenic projectors were used to perform a PDWF Wannierization. (d) Scatter plot of η_2 vs. η_2^{SOC}, showing that regardless of whether SOC-weighting is applied, the two band distances are approximately the same for most systems.

II. BAND DISTANCE COMPARISION

To complement the results shown in the main text, we also analyzed the band distance η_1 with lower energy window, and the maximum band distance η_0^{max}. Results are shown in Supplementary Fig. 2 and Fig. 3, respectively. Also when using these metrics, we obtain a significant reduction in band distance after introducing external hydrogenic AOs, for all pseudopotential libraries and both when considering SOC or not.

A. Band distance with lower energy window

We set the cut-off energy to $E_F + 1$ eV for metals and CBM + 1 eV for insulators or semiconductors. This energy window also lies above the Fermi level (or CBM), thus encompassing the entire valence band and a portion of the conduction bands. Results are shown in Supplementary Fig. 2.

Specifically, for materials calculated with the `PseudoDojo` library (panels a and b), the largest value of η_1 across the dataset is reduced from 237.22 meV to 10.53 meV after introducing external hydrogenic AOs, and the number of materials with $\eta_1 > 20$ meV is reduced from 35 to 0; for materials calculated with `modified-pslibrary` (panels c and d), the maximal value of η_1 is reduced from 187.27 meV to 9.87 meV, and the number of materials with $\eta_1 >$ 20 meV is reduced from 15 to 0; for materials calculated with SSSP/PBE/efficiency v1.1 (panels e and f) and without SOC, the maximal value of η_1 is reduced from 297.39 meV to 13.30 meV, and the number of materials with $\eta_1 > 20$ meV is reduced from 6 to 0. All systems in our calculation set thus exhibit $\eta_1 < 15$ meV after introducing extrnal hydrogenic projectors.

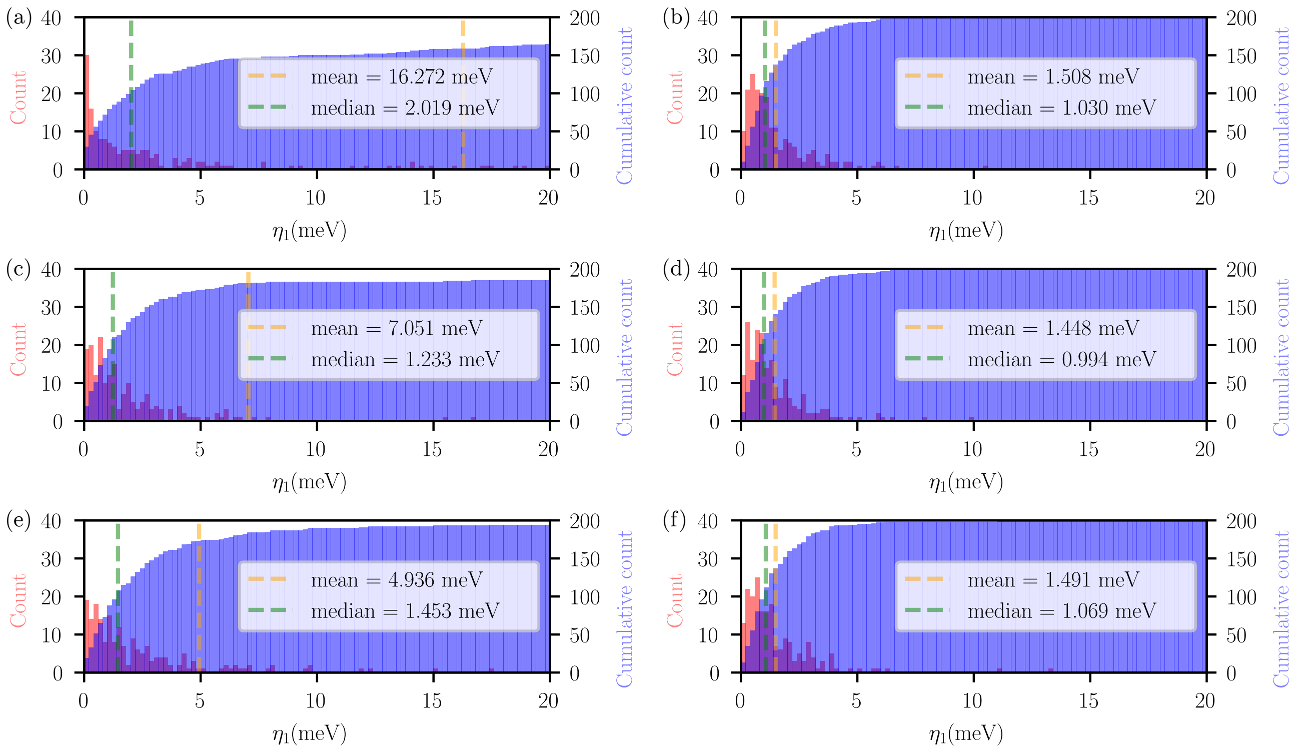

SUPPLEMENTARY FIG. 2. Histogram (red) and cumulative histogram (blue) of the maximum band distance η_1 for the 200 structures used in Ref. [1] using different projectors and pseudopotential libraries. All results were calculated with the PDWF method, and all frozen windows were set to $E_F + 1$ eV for metals, or to the conduction band minimum (CBM) + 1 eV for insulators. (a) PAOs from `PseudoDojo` (with SOC); (b) PAOs from `PseudoDojo` and external hydrogenic AOs (with SOC); (c) PAOs from `modified-pslibrary` (with SOC); (d) PAOs from `modified-pslibrary` and external hydrogenic AOs (with SOC); (e) PAOs from SSSP/PBE/efficiency v1.1 (without SOC); (f) PAOs from SSSP/PBE/efficiency v1.1 and external hydrogenic AOs (without SOC). The orange (green) vertical line is the mean (median) maximum band distance η_1, whose value(s) are shown in the legend of each panel.

B. Maximum band distance comparision

To further demonstrate the accuracy of Wannier interpolated band for the valence bands, we also analyze the more stringent maximum band distance η_0^{max}. Results are shown in Supplementary Fig. 3.

Specifically, for materials calculated with the `PseudoDojo` library (panels a and b), the largest value of η_0^{max} across the dataset is reduced from 2033.1 meV to 39.50 meV after introducing external hydrogenic AOs, and the number of materials with $\eta_0^{max} > 40$ meV (100 meV) is reduced from 56 (43) to 0 (0); for materials calculated with `modified-pslibrary` (panels c and d), the maximal value of η_0^{max} is reduced from 1880.6 meV to 42.78 meV, and the number of materials with $\eta_0^{max} > 40$ meV (100 meV) is reduced from 21 (14) to 1 (0); for materials calculated with SSSP/PBE/efficiency v1.1 (panels e and f) and without SOC, the maximal value of η_0^{max} is reduced from 901.1 meV to 41.99 meV, and the number of materials with $\eta_0^{max} > 40$ meV (100 meV) is reduced from 21 (10) to 2(0). These results demonstrate a significant enhancement in the robustness of the Wannier interpolated bands for the valence band.

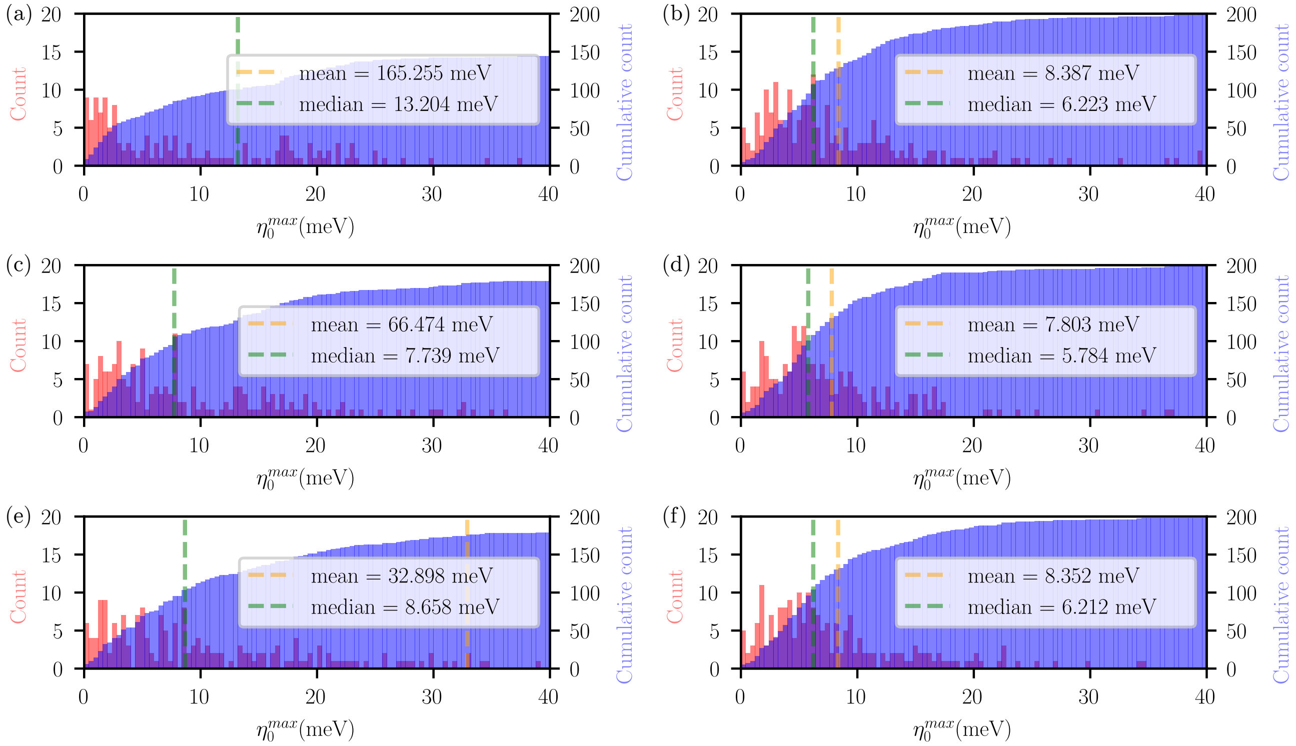

SUPPLEMENTARY FIG. 3. Histogram (red) and cumulative histogram (blue) of the maximum band distance η_0^{max} for the 200 structures used in Ref. [1] using different projectors and pseudopotential libraries. All results were calculated with the PDWF method, and all frozen windows were set to E_F eV for metals, or to the conduction band minimum (CBM) (not including any conduction band) for insulators. (a) PAOs from `PseudoDojo` (with SOC); (b) PAOs from `PseudoDojo` and external hydrogenic AOs (with SOC); (c) PAOs from `modified-pslibrary` (with SOC); (d) PAOs from `modified-pslibrary` and external hydrogenic AOs (with SOC); (e) PAOs from SSSP/PBE/efficiency v1.1 (without SOC); (f) PAOs from SSSP/PBE/efficiency v1.1 and external hydrogenic AOs (without SOC). The orange (green) vertical line is the mean (median) maximum band distance η_0^{max}, whose value(s) are shown in the legend of each panel.

III. BAND DISTANCE AFTER INTRODUCING EXTERNAL PROJECTORS

In Sec. II B of the main paper, we show that adding hydrogenic AOs can improve the performance of Wannier interpolation, especially in increasing the success rate in Supplementary Fig. 2. However, we observed that in regions with already very small band distance, the band distance might slightly increase after adding hydrogenic AOs. This effect is more pronounced when using `PseudoDojo`. To illustrate this, we have plotted the band distance η_2 before and after adding hydrogenic AOs in Supplementary Fig. 4. As a result, in the low-η_2 region, i.e. $\eta_2 \leq 5$ meV, there are instances where the external projector actually increases the band distance (see red crosses in Supplementary Fig. 4). However, since even despite this small increase, the band distance in these systems remains very small, the resulting Wannier interpolation is still of high quality. Fig. 4 also shows 6 structures with higher η_2^{ext} than η_2^{orig} in the region $\eta_2 > 5$ meV, but the increase is relatively small (< 2 meV) and thus negligible. We thus recommend to introduce by default the addition of external hydrogenic projectors, with the aim of improving the overall quality of Wannier interpolation for any input system in a general way. For systems negatively affected by this, a future workflow could incorporate a final *exclude projector* process to further optimize the results.

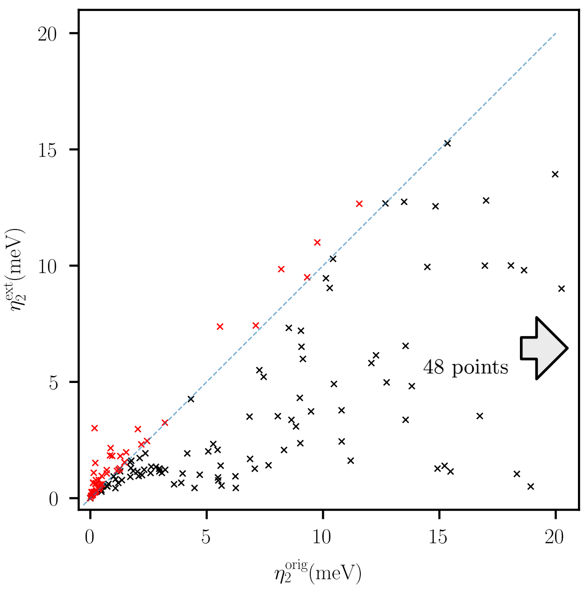

SUPPLEMENTARY FIG. 4. Scatter plot of the original band distance (η_2^{orig}) vs. the band distance after adding external projectors (η_2^{ext}) for materials calculated with `PseudoDojo`. The red crosses indicate systems for which the band distance increases after introducing external projectors (i.e., $\eta_2^{\text{ext}} > \eta_2^{\text{orig}}$). We note that for most of these points η_2^{ext} remains < 4 meV, and in general the addition of external projectors has a minimal impact on the overall robustness. 48 structures with large η_2^{orig} are not shown in the figure.

IV. ADDITIONAL COMPARISONS BETWEEN APPROACHES FOR DISENTANGLEMENT AND INITIAL PROJECTIONS

We present in Supplementary Fig. 5 the comparison between PAO projectors only and PAO with external hydrogenic AO projectors. At variance with Fig. 5 in the main text, the number of projectors is not the same in all panels. The results of Supplementary Fig. 5 shows that, whether using ED or PDWF, the introduction of external hydrogenic AOs can significantly enhance the robustness and accuracy of the Wannier interpolation.

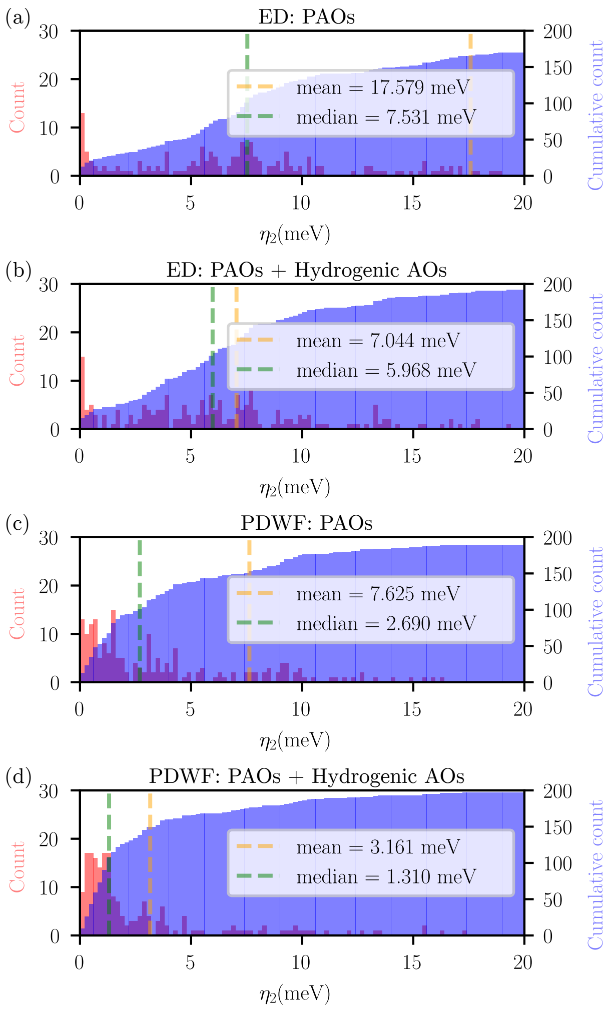

SUPPLEMENTARY FIG. 5. Histogram (red) and cumulative histogram (blue) of the band distance η_2 for the 200 structures used in Ref. [1] using different algorithms and projectors (without SOC). All frozen windows were set to E_F+2 eV for metals, or CBM + 2 eV for gapped systems (insulators). (a) ED with PAOs; (b) ED with PAOs and external hydrogenic AOs; (c) PDWF with PAOs (same as Ref. [1]); and (d) PDWF with PAOs and external hydrogenic AOs. The orange (green) vertical line is the mean (median) band distance η_2, whose value(s) are shown in the legend of each panel.

V. ORTHONORMALIZATION STRATEGY

As discussed in the main text, using a Löwdin orthonormalization on the complete set of the projectors derived from the PAOs in the pseudopotential and from the external hydrogenic AOs is detrimental, as the (chemically accurate) PAOs in the pseudopotential get mixed and thus distorted during orthogonalization.

Instead, we recommend to apply Löwdin orthonormalization separately to the projectors derived from the PAOs in the pseudopotential and those from the external hydrogenic AOs. Then, we perform Gram–Schmidt orthonormalization on the hydrogenic AOs projectors only, using the PAO projectors as the reference basis. In our implementation, we perform the two Löwdin orthonormalization steps in a single matrix operation, and adopt the modified Gram–Schmidt algorithm for improved numerical precision. This section provides a brief overview of the underlying implementation details.

Löwdin orthonormalization Suppose we have a complex matrix $A \in \mathbb{Z}_{m\times n}$, the Löwdin orthonormalization converts the columns of A into a set of orthonormal vectors that are as close as possible to the original columns. The columns of A should be linearly independent, thus requiring $m \geq n$. This is achieved in several steps:

1. compute the overlap matrix $O = A^\dagger A$, which is Hermitian by construction;
2. perform an eigen-decomposition of the overlap matrix, $O = PDP^{-1}$; the eigenvalues $D = \mathrm{diag}(\lambda_1, \ldots, \lambda_n)$ are real;
3. take the inverse square root of D, and create a new diagonal matrix $S = \mathrm{diag}(\frac{1}{\sqrt{\lambda_1}}, \ldots, \frac{1}{\sqrt{\lambda_n}})$, and a new matrix $U = PSP^{-1}$;
4. the Löwdin orthonormalized matrix is $B = AU$. B is semi-unitary since $B^\dagger B = (PS^\dagger P^\dagger A^\dagger)(APSP^{-1}) = PS^\dagger P^\dagger OPSP^{-1} = PS^\dagger DSP^{-1} = PP^{-1} = 1_{n\times n}$.

Suppose now that we have a block-diagonal matrix $A = \begin{bmatrix} A_1 & 0 \\ 0 & A_2 \end{bmatrix}$, then the overlap matrix is $O = \begin{bmatrix} A_1^\dagger A_1 & 0 \\ 0 & A_2^\dagger A_2 \end{bmatrix}$. The eigen decoposition of O is $O = PDP^{-1}$. It can be trivially proved that P and D can be arranged as block-diagonal matrices that consist of the eigenvectors and eigenvalues of A_1 and A_2. Therefore, the construction of U in step 3 is equivalent to two separate constructions of U_1 and U_2 for A_1 and A_2, and the multiplication of U_1 and U_2 is applied separately to A_1 and A_2. Thus, Löwdin orthonormalization of a block-diagonal matrix is equivalent to two separate Löwdin orthonormalizations of the submatrices.

In our case, this means that the Löwdin orthonormalization of the two sets of projectors, PAOs and hydrogenic AOs, can be combined together in a *one-shot* Löwdin orthonormalization. Special care needs to be taken to the case of ultrasoft pseudopotentials (USPP)—the inner product between two Bloch wave functions requires a Hermitian overlap operator $\hat{S}$ such that the orthonormalization condition becomes $\langle\psi_i|\hat{S}|\psi_j\rangle = \delta_{ij}$. For non-USPP cases, one can simply take $\hat{S} = \hat{I}$.

We have $n = n_P + n_H$ projectors, of which n_P are from Pseudo Atomic Orbitals (PAOs) and n_H from external hydrogenic AOs. The projector matrix can be separated into two blocks for the two sets of projectors,

$$A = \begin{bmatrix} A_P & A_H \end{bmatrix},$$

which is a $n_{\rm PW} \times n$ matrix, where $n_{\rm PW}$ is the number of plane waves in the basis set, and A_P and A_H are the projector matrices of PAOs and hydrogenic AOs, respectively. The overlap matrix is

$$O = A^\dagger \hat{S} A = \begin{bmatrix} A_P^\dagger \hat{S} A_P & A_P^\dagger \hat{S} A_H \\ A_H^\dagger \hat{S} A_P & A_H^\dagger \hat{S} A_H \end{bmatrix}.$$

However, we do *not* use the conventional Löwdin orthonormalization on the full set of n projectors, as the procedure would also change the PAOs. So we first set the $A_H^\dagger \hat{S} A_P$ and $A_P^\dagger \hat{S} A_H$ to 0 in the overlap matrix, and then perform a Löwdin orthonormalization. According to previous paragraphs, the rotation matrix U of the Löwdin orthonormalization can actually be written as

$$U = \begin{bmatrix} U_P & 0 \\ 0 & U_H \end{bmatrix},$$

which are two separate rotations for the two sets of projectors. The orthonormalized projectors become

$$B = \begin{bmatrix} A_P U_P & A_H U_H \end{bmatrix},$$

and

$$\hat{S} B = \begin{bmatrix} \hat{S} A_P U_P & \hat{S} A_H U_H \end{bmatrix}.$$

Gram–Schmidt orthonormalization In the previous Section on Löwdin orthonormalization, we have ensured that the PAO projectors $A_P U_P$ and the hydrogenic AOs projectors $A_H U_H$ are both orthonormal internally. However, the $A_H U_H$ projectors are not orthogonal to the $A_P U_P$ projectors, i.e., the off-diagonal part of the overlap matrix $\langle B, \hat{S} B \rangle$ is not 0. Therefore, we choose to apply an extra Gram–Schmidt (GS) process to orthonormalize the projector matrix, aiming to keep the PAOs unchanged. We therefore consider the projectors from PAOs frozen, while hydrogenic AOs are free to change. We show the detailed steps of our implementation in the following algorithm, which is based on modified GS algorithm (which has lower numerical errors than the classical GS algorithm):

Algorithm 1 Orthonormalization of PAOs and hydrogenic AOs in `pw2wannier90.x`

$O = A^\dagger \hat{S} A$
set off-diagonal part of O to 0
$D, P = \text{eigenvalue}(O), \text{eigenvector}(O)$
$U = P \cdot \text{diag}(D)^{-1/2} \cdot P^{-1}$
$B = AU$
$SB = \hat{S} AU$
for projector j **not in** frozen projectors **do**
  for projector i **in** frozen projectors **do**
    $r = \langle B[:, i], SB[:, j] \rangle$
    $B[:, j] = B[:, j] - r \cdot B[:, i]$
    $SB[:, j] = SB[:, j] - r \cdot SB[:, i]$
  end for
  $r = \langle B[:, j], SB[:, j] \rangle$
  $B[:, j] = B[:, j]/r$
  $SB[:, j] = SB[:, j]/r$
  set projector j frozen
end for

The final projectors SB can be multiplied with the Bloch wave functions to obtain the initial gauge matrices for Wannierization.

In summary, our modified Löwdin orthonormalization ensures that the set of PAO projectors and the set of hydrogenic AO projectors are both orthonormal internally, and the modified Gram–Schmidt orthonormalization ensures the hydrogenic AOs are orthonormal to the PAOs, i.e., the part of gauge matrices from PAO projections are kept unchanged compared to calculations without hydrogenic AOs, while the hydrogenic AOs are complementary orbitals orthonormal to the PAOs, providing a more complete basis set for Wannierization when PAOs are not sufficient.

The code is implemented in the `pw2wannier90.x` code of the Quantum ESPRESSO package starting from version 7.5.

VI. MAGNETIC STRUCTURES

With the goal of selecting a representative set of materials with a diversity of magnetic moment configurations, we selected several small- to medium-scale collinear and non-collinear magnetic structures from the MAGNDATA database [2]. These are graphically shown in Supplementary Fig. 6 and listed in Supplementary Table I.

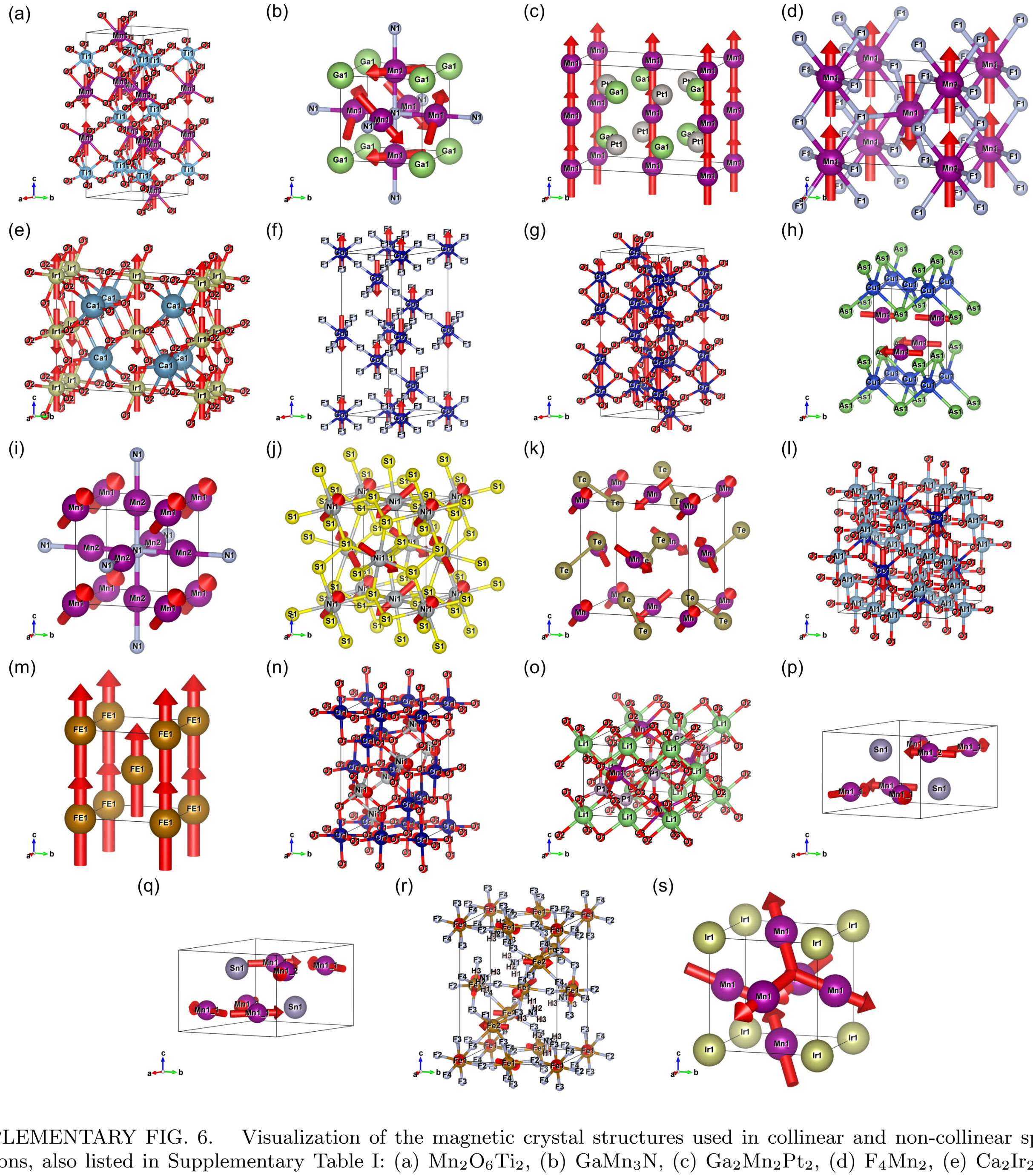

SUPPLEMENTARY FIG. 6. Visualization of the magnetic crystal structures used in collinear and non-collinear spin calculations, also listed in Supplementary Table I: (a) $Mn_2O_6Ti_2$, (b) $GaMn_3N$, (c) $Ga_2Mn_2Pt_2$, (d) F_4Mn_2, (e) $Ca_2Ir_2O_6$, (f) Co_2F_6, (g) Cr_4O_6, (h) $As_2Cu_2Mn_2$, (i) Mn_4N, (j) Ni_4S_8, (k) Mn_4Te_8, (l) $Al_4Co_2O_8$, (m) Fe, (n) $Cr_4Ni_2O_8$, (o) $Li_4Mn_4O_{16}P_4$, (p) Mn_6Sn_2, (q) Mn_6Sn_2, (r) $F_{24}Fe_8H_{16}N_4$, (s) $IrMn_3$. Magnetic moments are represented by red arrows.

SUPPLEMENTARY TABLE I. Magnetic structures used in collinear and non-collinear spin calculations.

Label	Formula	Collinear calculation	Non-collinear calculation
a	$Mn_2O_6Ti_2$	✓	✓
b	$GaMn_3N$	✓	
c	$Ga_2Mn_2Pt_2$	✓	✓
d	F_4Mn_2	✓	✓
e	$Ca_2Ir_2O_6$	✓	✓
f	Co_2F_6	✓	✓
g	Cr_4O_6	✓	✓
h	$As_2Cu_2Mn_2$	✓	✓
i	Mn_4N	✓	✓
j	Ni_4S_8	✓	✓
k	Mn_4Te_8	✓	✓
l	$Al_4Co_2O_8$	✓	✓
m	Fe	✓	✓
n	$Cr_4Ni_2O_8$		✓
o	$Li_4Mn_4O_{16}P_4$	✓	✓
p	Mn_6Sn_2		✓
q	Mn_6Sn_2		✓
r	$F_{24}Fe_8H_{16}N_4$	✓	
s	$IrMn_3$	✓	

VII. REQUIRED PROJECTORS SET FOR A ROBUST PDWF WANNIERIZATION

In the main text, we employed a relatively aggressive strategy (see Section II B in the main text for detailed rules) to define a set of projectors that stabilize most PDWFs. These orbitals are listed in Supplementary Table II. We stress that, however, in practical applications this setting may result in an excessive number of projectors for certain structures. Thus, users are encouraged to select an appropriate projector set based on the specific system of interest.

SUPPLEMENTARY TABLE II: Required orbitals for a robust PDWF Wannierization.

Z	Element	Required orbitals
1	H	$1s$
2	He	$1s$
3	Li	$2s$ $2p$
4	Be	$2s$ $2p$
5	B	$2s$ $2p$
6	C	$2s$ $2p$
7	N	$2s$ $2p$
8	O	$2s$ $2p$
9	F	$2s$ $2p$
10	Ne	$2s$ $2p$
11	Na	$3s$ $3p$
12	Mg	$3s$ $3p$
13	Al	$3s$ $3p$
14	Si	$3s$ $3p$
15	P	$3s$ $3p$
16	S	$3s$ $3p$
17	Cl	$3s$ $3p$
18	Ar	$3s$ $3p$
19	K	$4s$ $3d$
20	Ca	$4s$ $3d$
21	Sc	$4s$ $4p$ $3d$
22	Ti	$4s$ $4p$ $3d$
23	V	$4s$ $4p$ $3d$
24	Cr	$4s$ $4p$ $3d$
25	Mn	$4s$ $4p$ $3d$
26	Fe	$4s$ $4p$ $3d$
27	Co	$4s$ $4p$ $3d$
28	Ni	$4s$ $4p$ $3d$
29	Cu	$4s$ $4p$ $3d$
30	Zn	$4s$ $4p$ $3d$
31	Ga	$4s$ $4p$
32	Ge	$4s$ $4p$
33	As	$4s$ $4p$
34	Se	$4s$ $4p$
35	Br	$4s$ $4p$
36	Kr	$4s$ $4p$
37	Rb	$5s$ $4d$
38	Sr	$5s$ $4d$
39	Y	$5s$ $5p$ $4d$
40	Zr	$5s$ $5p$ $4d$
41	Nb	$5s$ $5p$ $4d$
42	Mo	$5s$ $5p$ $4d$
43	Tc	$5s$ $5p$ $4d$
44	Ru	$5s$ $5p$ $4d$
45	Rh	$5s$ $5p$ $4d$
46	Pd	$5s$ $5p$ $4d$

Z	Element	Required orbitals
47	Ag	$5s$ $5p$ $4d$
48	Cd	$5s$ $5p$ $4d$
49	In	$5s$ $5p$
50	Sn	$5s$ $5p$
51	Sb	$5s$ $5p$
52	Te	$5s$ $5p$
53	I	$5s$ $5p$
54	Xe	$5s$ $5p$
55	Cs	$6s$ $5d$
56	Ba	$6s$ $5d$
72	Hf	$6s$ $6p$ $5d$
73	Ta	$6s$ $6p$ $5d$
74	W	$6s$ $6p$ $5d$
75	Re	$6s$ $6p$ $5d$
76	Os	$6s$ $6p$ $5d$
77	Ir	$6s$ $6p$ $5d$
78	Pt	$6s$ $6p$ $5d$
79	Au	$6s$ $6p$ $5d$
80	Hg	$6s$ $6p$ $5d$
81	Tl	$6s$ $6p$
82	Pb	$6s$ $6p$
83	Bi	$6s$ $6p$
84	Po	$6s$ $6p$
85	At	$6s$ $6p$
86	Rn	$6s$ $6p$

VIII. DETAILED INFORMATION ON THE PROJECTORS USED IN THIS WORK

Based on the radial expression of hydrogenic AOs (see Table I in main text for details), we obtained external projectors using different approaches, including orthonormalization fitting (detailed see Section II B in the main text) and all-hydrogenic fitting, which is the method to generate the projectors for "corrected hydrogenic AOs". The latter involves fitting the electron in the innermost shell to the pseudopotential PAOs while applying orthogonalization methods as much as possible to assign α to outer hydrogenic AOs. For missing AOs without inner shells, where therefore there is no orthogonality condition for the radial part to satisfy, we consider PAOs from OpenMX [3] and fit them accordingly to obtain the α values.

The values for the various pseudopotential libraries used in the main text, as well as using different orthogonalization fitting schemes, are reported in Supplementary Tables III, IV and V. The choice of α for the `modified-pslibrary` set (whose choice of pseudopotentials is reported in Supplementary Table VI) is reported in Supplementary Table VII.

In the Source column of the tables, "Pseudopotential" means that the orbital is simply taken from the PAO from the pseudopotential file, "Orthogonalization" means that the value is obtained by achieving orthogonality with the underlying PAO with same angular momentum l, while "OpenMX Fitting" or "Pseudopotential Fitting" indicate that the value has been obtained by fitting the corresponding atomic orbital from the OpenMX dataset [3] or from the pseudopotential PAOs. α values are in unit of bohr^{-1}.

Finally, to facilitate users in generating external projectors for other pseudopotential libraries, we provide in Supplementary Table VIII the α values for all hydrogenic AOs that we fitted in this work using `OpenMX`. This table allows users to use data directly from the table for projector generation. The recommended procedure to determine the α values for a new pseudopotential library is the following. First, use our script in the AiiDA-Wannier90-Workflows repository to generate external hydrogenic AOs based on the pseudopotential PAOs. Then, for missing projectors with nodes, employ orthogonalization fitting with the inner PAOs from pseudopotential to ensure greater accuracy. Finally, for any remaining projectors without nodes, use the value from Supplementary Table VIII.

SUPPLEMENTARY TABLE III: Values of α and minimal set of orbitals for the pseudopotentials of the SSSP PBE Efficiency v1.1 with orthonormalization fitting.

Z	Element	Orbital	l	Source	α(bohr^{-1})
1	H	1s	0	Pseudopotential	—
2	He	1s	0	Pseudopotential	—
3	Li	1s	0	Pseudopotential	—
		2s	0	Pseudopotential	—
		2p	1	Pseudopotential	—
4	Be	1s	0	Pseudopotential	—
		2s	0	Pseudopotential	—
		2p	1	Pseudopotential	—
5	B	2s	0	Pseudopotential	—
		2p	1	Pseudopotential	—
6	C	2s	0	Pseudopotential	—
		2p	1	Pseudopotential	—
7	N	2s	0	Pseudopotential	—
		2p	1	Pseudopotential	—
8	O	2s	0	Pseudopotential	—
		2p	1	Pseudopotential	—
9	F	2s	0	Pseudopotential	—
		2p	1	Pseudopotential	—
10	Ne	2s	0	Pseudopotential	—
		2p	1	Pseudopotential	—
11	Na	2s	0	Pseudopotential	—
		3s	0	Pseudopotential	—
		2p	1	Pseudopotential	—
		3p	1	Orthogonalization	4.500
12	Mg	3s	0	Pseudopotential	—
		3p	1	Pseudopotential	—
13	Al	3s	0	Pseudopotential	—
		3p	1	Pseudopotential	—
14	Si	3s	0	Pseudopotential	—
		3p	1	Pseudopotential	—
15	P	3s	0	Pseudopotential	—
		3p	1	Pseudopotential	—
16	S	3s	0	Pseudopotential	—
		3p	1	Pseudopotential	—
17	Cl	3s	0	Pseudopotential	—
		3p	1	Pseudopotential	—
18	Ar	3s	0	Pseudopotential	—
		3p	1	Pseudopotential	—
19	K	3s	0	Pseudopotential	—
		4s	0	Pseudopotential	—
		3p	1	Pseudopotential	—
		4p	1	Pseudopotential	—
		3d	2	OpenMX Fitting	2.727
20	Ca	3s	0	Pseudopotential	—
		4s	0	Pseudopotential	—
		3p	1	Pseudopotential	—
		3d	2	Pseudopotential	—
21	Sc	3s	0	Pseudopotential	—
		4s	0	Pseudopotential	—
		3p	1	Pseudopotential	—
		3d	2	Pseudopotential	—
		4p	1	Orthogonalization	4.200
22	Ti	3s	0	Pseudopotential	—
		4s	0	Pseudopotential	—
		3p	1	Pseudopotential	—
		3d	2	Pseudopotential	—
		4p	1	Orthogonalization	4.400
23	V	3s	0	Pseudopotential	—
		4s	0	Pseudopotential	—
		3p	1	Pseudopotential	—
		3d	2	Pseudopotential	—
		4p	1	Orthogonalization	4.600
24	Cr	3s	0	Pseudopotential	—
		4s	0	Pseudopotential	—
		3p	1	Pseudopotential	—
		3d	2	Pseudopotential	—
		4p	1	Orthogonalization	4.900
25	Mn	3s	0	Pseudopotential	—
		4s	0	Pseudopotential	—
		3p	1	Pseudopotential	—
		4p	1	Pseudopotential	—
		3d	2	Pseudopotential	—
26	Fe	3s	0	Pseudopotential	—
		4s	0	Pseudopotential	—
		3p	1	Pseudopotential	—
		4p	1	Pseudopotential	—
		3d	2	Pseudopotential	—
27	Co	3s	0	Pseudopotential	—
		4s	0	Pseudopotential	—
		3p	1	Pseudopotential	—
		4p	1	Pseudopotential	—
		3d	2	Pseudopotential	—
28	Ni	3s	0	Pseudopotential	—
		4s	0	Pseudopotential	—
		3p	1	Pseudopotential	—
		4p	1	Pseudopotential	—
		3d	2	Pseudopotential	—
29	Cu	3s	0	Pseudopotential	—
		4s	0	Pseudopotential	—
		3p	1	Pseudopotential	—
		4p	1	Pseudopotential	—
		3d	2	Pseudopotential	—
30	Zn	3s	0	Pseudopotential	—
		4s	0	Pseudopotential	—
		3p	1	Pseudopotential	—
		4p	1	Pseudopotential	—
		3d	2	Pseudopotential	—
31	Ga	4s	0	Pseudopotential	—
		4p	1	Pseudopotential	—
		3d	2	Pseudopotential	—
32	Ge	4s	0	Pseudopotential	—
		4p	1	Pseudopotential	—
		3d	2	Pseudopotential	—
33	As	4s	0	Pseudopotential	—
		4p	1	Pseudopotential	—
34	Se	4s	0	Pseudopotential	—
		4p	1	Pseudopotential	—
35	Br	4s	0	Pseudopotential	—
		4p	1	Pseudopotential	—
36	Kr	4s	0	Pseudopotential	—
		4p	1	Pseudopotential	—
37	Rb	4s	0	Pseudopotential	—
		5s	0	Pseudopotential	—
		4p	1	Pseudopotential	—
		4d	2	OpenMX Fitting	2.197

Z	Element	Orbital	l	Source	α(bohr^{-1})
38	Sr	$4s$	0	Pseudopotential	—
		$5s$	0	Pseudopotential	—
		$4p$	1	Pseudopotential	—
		$5p$	1	Pseudopotential	—
		$4d$	2	Pseudopotential	—
39	Y	$4s$	0	Pseudopotential	—
		$5s$	0	Pseudopotential	—
		$4p$	1	Pseudopotential	—
		$5p$	1	Pseudopotential	—
		$4d$	2	Pseudopotential	—
40	Zr	$4s$	0	Pseudopotential	—
		$5s$	0	Pseudopotential	—
		$4p$	1	Pseudopotential	—
		$5p$	1	Pseudopotential	—
		$4d$	2	Pseudopotential	—
41	Nb	$4s$	0	Pseudopotential	—
		$5s$	0	Pseudopotential	—
		$4p$	1	Pseudopotential	—
		$4d$	2	Pseudopotential	—
		$5p$	1	Orthogonalization	4.100
42	Mo	$4s$	0	Pseudopotential	—
		$5s$	0	Pseudopotential	—
		$4p$	1	Pseudopotential	—
		$4d$	2	Pseudopotential	—
		$5p$	1	Orthogonalization	4.300
43	Tc	$4s$	0	Pseudopotential	—
		$5s$	0	Pseudopotential	—
		$4p$	1	Pseudopotential	—
		$4d$	2	Pseudopotential	—
		$5p$	1	Orthogonalization	4.400
44	Ru	$4s$	0	Pseudopotential	—
		$5s$	0	Pseudopotential	—
		$4p$	1	Pseudopotential	—
		$4d$	2	Pseudopotential	—
		$5p$	1	Orthogonalization	4.600
45	Rh	$4s$	0	Pseudopotential	—
		$5s$	0	Pseudopotential	—
		$4p$	1	Pseudopotential	—
		$4d$	2	Pseudopotential	—
		$5p$	1	Orthogonalization	4.800
46	Pd	$4s$	0	Pseudopotential	—
		$5s$	0	Pseudopotential	—
		$4p$	1	Pseudopotential	—
		$4d$	2	Pseudopotential	—
		$5p$	1	Orthogonalization	5.000
47	Ag	$4s$	0	Pseudopotential	—
		$5s$	0	Pseudopotential	—
		$4p$	1	Pseudopotential	—
		$4d$	2	Pseudopotential	—
		$5p$	1	Orthogonalization	5.100
48	Cd	$5s$	0	Pseudopotential	—
		$5p$	1	Pseudopotential	—
		$4d$	2	Pseudopotential	—
49	In	$5s$	0	Pseudopotential	—
		$5p$	1	Pseudopotential	—
		$4d$	2	Pseudopotential	—
50	Sn	$5s$	0	Pseudopotential	—
		$5p$	1	Pseudopotential	—
		$4d$	2	Pseudopotential	—
51	Sb	$5s$	0	Pseudopotential	—
		$5p$	1	Pseudopotential	—
		$4d$	2	Pseudopotential	—

Z	Element	Orbital	l	Source	α(bohr^{-1})
52	Te	$5s$	0	Pseudopotential	—
		$5p$	1	Pseudopotential	—
53	I	$5s$	0	Pseudopotential	—
		$5p$	1	Pseudopotential	—
54	Xe	$5s$	0	Pseudopotential	—
		$5p$	1	Pseudopotential	—
		$4d$	2	Pseudopotential	—
55	Cs	$5s$	0	Pseudopotential	—
		$6s$	0	Pseudopotential	—
		$5p$	1	Pseudopotential	—
		$6p$	1	Pseudopotential	—
		$5d$	2	Pseudopotential	—
56	Ba	$5s$	0	Pseudopotential	—
		$6s$	0	Pseudopotential	—
		$5p$	1	Pseudopotential	—
		$5d$	2	OpenMX Fitting	3.039
72	Hf	$5s$	0	Pseudopotential	—
		$6s$	0	Pseudopotential	—
		$5p$	1	Pseudopotential	—
		$5d$	2	Pseudopotential	—
		$6p$	1	Orthogonalization	3.900
73	Ta	$5s$	0	Pseudopotential	—
		$6s$	0	Pseudopotential	—
		$5p$	1	Pseudopotential	—
		$6p$	1	Pseudopotential	—
		$5d$	2	Pseudopotential	—
74	W	$5s$	0	Pseudopotential	—
		$6s$	0	Pseudopotential	—
		$5p$	1	Pseudopotential	—
		$6p$	1	Pseudopotential	—
		$5d$	2	Pseudopotential	—
75	Re	$5s$	0	Pseudopotential	—
		$6s$	0	Pseudopotential	—
		$5p$	1	Pseudopotential	—
		$6p$	1	Pseudopotential	—
		$5d$	2	Pseudopotential	—
76	Os	$5s$	0	Pseudopotential	—
		$6s$	0	Pseudopotential	—
		$5p$	1	Pseudopotential	—
		$6p$	1	Pseudopotential	—
		$5d$	2	Pseudopotential	—
77	Ir	$6s$	0	Pseudopotential	—
		$5p$	1	Pseudopotential	—
		$6p$	1	Pseudopotential	—
		$5d$	2	Pseudopotential	—
78	Pt	$6s$	0	Pseudopotential	—
		$5p$	1	Pseudopotential	—
		$6p$	1	Pseudopotential	—
		$5d$	2	Pseudopotential	—
79	Au	$5s$	0	Pseudopotential	—
		$6s$	0	Pseudopotential	—
		$5p$	1	Pseudopotential	—
		$5d$	2	Pseudopotential	—
		$6p$	1	Orthogonalization	5.000
80	Hg	$5s$	0	Pseudopotential	—
		$6s$	0	Pseudopotential	—
		$5p$	1	Pseudopotential	—
		$5d$	2	Pseudopotential	—
		$6p$	1	Orthogonalization	5.100
81	Tl	$6s$	0	Pseudopotential	—
		$6p$	1	Pseudopotential	—
		$5d$	2	Pseudopotential	—

Z	Element	Orbital	l	Source	α(bohr^{-1})
82	Pb	$6s$	0	Pseudopotential	—
		$6p$	1	Pseudopotential	—
		$5d$	2	Pseudopotential	—
83	Bi	$6s$	0	Pseudopotential	—
		$6p$	1	Pseudopotential	—
		$5d$	2	Pseudopotential	—
84	Po	$6s$	0	Pseudopotential	—
		$6p$	1	Pseudopotential	—
		$5d$	2	Pseudopotential	—
86	Rn	$6s$	0	Pseudopotential	—
		$6p$	1	Pseudopotential	—
		$5d$	2	Pseudopotential	—

SUPPLEMENTARY TABLE IV: Values of α and minimal set of orbitals for the pseudopotentials of the SSSP PBE Efficiency v1.1 with all-hydrogenic fitting.

Z	Element	Orbital	l	Source	α(bohr^{-1})
1	H	$1s$	0	Pseudopotential Fitting	0.910
2	He	$1s$	0	Pseudopotential Fitting	1.522
3	Li	$1s$	0	Pseudopotential Fitting	1.252
		$2s$	0	Orthogonalization	1.252
		$2p$	1	Pseudopotential Fitting	1.205
4	Be	$1s$	0	Pseudopotential Fitting	0.702
		$2s$	0	Orthogonalization	0.702
		$2p$	1	Pseudopotential Fitting	1.612
5	B	$2s$	0	Pseudopotential Fitting	0.614
		$2p$	1	Pseudopotential Fitting	2.283
6	C	$2s$	0	Pseudopotential Fitting	0.863
		$2p$	1	Pseudopotential Fitting	2.834
7	N	$2s$	0	Pseudopotential Fitting	1.109
		$2p$	1	Pseudopotential Fitting	3.195
8	O	$2s$	0	Pseudopotential Fitting	1.287
		$2p$	1	Pseudopotential Fitting	3.564
9	F	$2s$	0	Pseudopotential Fitting	1.403
		$2p$	1	Pseudopotential Fitting	3.476
10	Ne	$2s$	0	Pseudopotential Fitting	1.565
		$2p$	1	Pseudopotential Fitting	4.326
11	Na	$2s$	0	Pseudopotential Fitting	1.516
		$3s$	0	Orthogonalization	1.516
		$2p$	1	Pseudopotential Fitting	3.456
		$3p$	1	Orthogonalization	3.456
12	Mg	$3s$	0	Pseudopotential Fitting	0.414
		$3p$	1	Pseudopotential Fitting	1.078
13	Al	$3s$	0	Pseudopotential Fitting	0.478
		$3p$	1	Pseudopotential Fitting	1.412
14	Si	$3s$	0	Pseudopotential Fitting	0.595
		$3p$	1	Pseudopotential Fitting	1.752
15	P	$3s$	0	Pseudopotential Fitting	0.744
		$3p$	1	Pseudopotential Fitting	2.117
16	S	$3s$	0	Pseudopotential Fitting	0.722
		$3p$	1	Pseudopotential Fitting	2.347
17	Cl	$3s$	0	Pseudopotential Fitting	0.797
		$3p$	1	Pseudopotential Fitting	2.637
18	Ar	$3s$	0	Pseudopotential Fitting	1.057
		$3p$	1	Pseudopotential Fitting	2.980
19	K	$3s$	0	Pseudopotential Fitting	1.139
		$4s$	0	Orthogonalization	1.139
		$3p$	1	Pseudopotential Fitting	3.410
		$4p$	1	Orthogonalization	3.410
		$3d$	2	OpenMX Fitting	2.727
20	Ca	$3s$	0	Pseudopotential Fitting	1.266
		$4s$	0	Orthogonalization	1.266
		$3p$	1	Pseudopotential Fitting	3.257
		$3d$	2	Pseudopotential Fitting	4.172
21	Sc	$3s$	0	Pseudopotential Fitting	1.449
		$4s$	0	Orthogonalization	1.449
		$3p$	1	Pseudopotential Fitting	4.074
		$3d$	2	Pseudopotential Fitting	5.810
		$4p$	1	Orthogonalization	4.074
22	Ti	$3s$	0	Pseudopotential Fitting	1.262
		$4s$	0	Orthogonalization	1.262
		$3p$	1	Pseudopotential Fitting	3.514
		$3d$	2	Pseudopotential Fitting	5.725
		$4p$	1	Orthogonalization	3.514

Z	Element	Orbital	l	Source	α(bohr^{-1})
		$3s$	0	Pseudopotential Fitting	1.541
		$4s$	0	Orthogonalization	1.541
23	V	$3p$	1	Pseudopotential Fitting	3.861
		$3d$	2	Pseudopotential Fitting	5.662
		$4p$	1	Orthogonalization	3.861
		$3s$	0	Pseudopotential Fitting	1.598
		$4s$	0	Orthogonalization	1.598
24	Cr	$3p$	1	Pseudopotential Fitting	3.949
		$3d$	2	Pseudopotential Fitting	5.999
		$4p$	1	Orthogonalization	3.949
		$3s$	0	Pseudopotential Fitting	1.655
		$4s$	0	Orthogonalization	1.655
25	Mn	$3p$	1	Pseudopotential Fitting	4.061
		$4p$	1	Orthogonalization	4.061
		$3d$	2	Pseudopotential Fitting	5.857
		$3s$	0	Pseudopotential Fitting	1.804
		$4s$	0	Orthogonalization	1.804
26	Fe	$3p$	1	Pseudopotential Fitting	4.925
		$4p$	1	Orthogonalization	4.925
		$3d$	2	Pseudopotential Fitting	5.248
		$3s$	0	Pseudopotential Fitting	1.656
		$4s$	0	Orthogonalization	1.656
27	Co	$3p$	1	Pseudopotential Fitting	3.888
		$4p$	1	Orthogonalization	3.888
		$3d$	2	Pseudopotential Fitting	6.165
		$3s$	0	Pseudopotential Fitting	1.746
		$4s$	0	Orthogonalization	1.746
28	Ni	$3p$	1	Pseudopotential Fitting	3.868
		$4p$	1	Orthogonalization	3.868
		$3d$	2	Pseudopotential Fitting	6.521
		$3s$	0	Pseudopotential Fitting	1.674
		$4s$	0	Orthogonalization	1.674
29	Cu	$3p$	1	Pseudopotential Fitting	3.477
		$4p$	1	Orthogonalization	3.477
		$3d$	2	Pseudopotential Fitting	6.327
		$3s$	0	Pseudopotential Fitting	1.676
		$4s$	0	Orthogonalization	1.676
30	Zn	$3p$	1	Pseudopotential Fitting	3.762
		$4p$	1	Orthogonalization	3.762
		$3d$	2	Pseudopotential Fitting	5.849
		$4s$	0	Pseudopotential Fitting	0.558
31	Ga	$4p$	1	Pseudopotential Fitting	1.484
		$3d$	2	Pseudopotential Fitting	5.166
		$4s$	0	Pseudopotential Fitting	0.607
32	Ge	$4p$	1	Pseudopotential Fitting	1.669
		$3d$	2	Pseudopotential Fitting	1.332
33	As	$4s$	0	Pseudopotential Fitting	0.710
		$4p$	1	Pseudopotential Fitting	1.984
34	Se	$4s$	0	Pseudopotential Fitting	0.668
		$4p$	1	Pseudopotential Fitting	2.068
35	Br	$4s$	0	Pseudopotential Fitting	0.713
		$4p$	1	Pseudopotential Fitting	2.267
36	Kr	$4s$	0	Pseudopotential Fitting	0.985
		$4p$	1	Pseudopotential Fitting	2.566
		$4s$	0	Pseudopotential Fitting	1.099
37	Rb	$5s$	0	Orthogonalization	1.099
		$4p$	1	Pseudopotential Fitting	2.894
		$4d$	2	OpenMX Fitting	2.197

Z	Element	Orbital	l	Source	α(bohr^{-1})
		$4s$	0	Pseudopotential Fitting	1.064
		$5s$	0	Orthogonalization	1.064
38	Sr	$4p$	1	Pseudopotential Fitting	3.045
		$5p$	1	Orthogonalization	3.045
		$4d$	2	Pseudopotential Fitting	3.121
		$4s$	0	Pseudopotential Fitting	1.164
		$5s$	0	Orthogonalization	1.164
39	Y	$4p$	1	Pseudopotential Fitting	3.058
		$5p$	1	Orthogonalization	3.058
		$4d$	2	Pseudopotential Fitting	3.940
		$4s$	0	Pseudopotential Fitting	1.266
		$5s$	0	Orthogonalization	1.266
40	Zr	$4p$	1	Pseudopotential Fitting	3.284
		$5p$	1	Orthogonalization	3.284
		$4d$	2	Pseudopotential Fitting	4.372
		$4s$	0	Pseudopotential Fitting	1.356
		$5s$	0	Orthogonalization	1.356
41	Nb	$4p$	1	Pseudopotential Fitting	3.700
		$4d$	2	Pseudopotential Fitting	5.157
		$5p$	1	Orthogonalization	3.700
		$4s$	0	Pseudopotential Fitting	1.456
		$5s$	0	Orthogonalization	1.456
42	Mo	$4p$	1	Pseudopotential Fitting	4.060
		$4d$	2	Pseudopotential Fitting	5.618
		$5p$	1	Orthogonalization	4.060
		$4s$	0	Pseudopotential Fitting	1.556
		$5s$	0	Orthogonalization	1.556
43	Tc	$4p$	1	Pseudopotential Fitting	4.215
		$4d$	2	Pseudopotential Fitting	5.984
		$5p$	1	Orthogonalization	4.215
		$4s$	0	Pseudopotential Fitting	1.638
		$5s$	0	Orthogonalization	1.638
44	Ru	$4p$	1	Pseudopotential Fitting	4.388
		$4d$	2	Pseudopotential Fitting	6.369
		$5p$	1	Orthogonalization	4.388
		$4s$	0	Pseudopotential Fitting	1.700
		$5s$	0	Orthogonalization	1.700
45	Rh	$4p$	1	Pseudopotential Fitting	4.505
		$4d$	2	Pseudopotential Fitting	6.670
		$5p$	1	Orthogonalization	4.505
		$4s$	0	Pseudopotential Fitting	1.767
		$5s$	0	Orthogonalization	1.767
46	Pd	$4p$	1	Pseudopotential Fitting	4.703
		$4d$	2	Pseudopotential Fitting	7.214
		$5p$	1	Orthogonalization	4.703
		$4s$	0	Pseudopotential Fitting	1.820
		$5s$	0	Orthogonalization	1.820
47	Ag	$4p$	1	Pseudopotential Fitting	4.835
		$4d$	2	Pseudopotential Fitting	7.455
		$5p$	1	Orthogonalization	4.835
		$5s$	0	Pseudopotential Fitting	0.428
48	Cd	$5p$	1	Pseudopotential Fitting	1.156
		$4d$	2	Pseudopotential Fitting	6.627
		$5s$	0	Pseudopotential Fitting	0.433
49	In	$5p$	1	Pseudopotential Fitting	1.320
		$4d$	2	Pseudopotential Fitting	6.051
		$5s$	0	Pseudopotential Fitting	0.313
50	Sn	$5p$	1	Pseudopotential Fitting	1.410
		$4d$	2	Pseudopotential Fitting	5.547
		$5s$	0	Pseudopotential Fitting	0.347
51	Sb	$5p$	1	Pseudopotential Fitting	1.557
		$4d$	2	Pseudopotential Fitting	6.769

Z	Element	Orbital	l	Source	$\alpha(\text{bohr}^{-1})$
52	Te	$5s$	0	Pseudopotential Fitting	0.606
		$5p$	1	Pseudopotential Fitting	1.731
53	I	$5s$	0	Pseudopotential Fitting	0.684
		$5p$	1	Pseudopotential Fitting	1.840
54	Xe	$5s$	0	Pseudopotential Fitting	0.802
		$5p$	1	Pseudopotential Fitting	2.134
		$4d$	2	Pseudopotential Fitting	9.378
55	Cs	$5s$	0	Pseudopotential Fitting	0.826
		$6s$	0	Orthogonalization	0.826
		$5p$	1	Pseudopotential Fitting	2.284
		$6p$	1	Orthogonalization	2.284
		$5d$	2	Pseudopotential Fitting	1.993
56	Ba	$5s$	0	Pseudopotential Fitting	0.956
		$6s$	0	Orthogonalization	0.956
		$5p$	1	Pseudopotential Fitting	2.625
		$5d$	2	OpenMX Fitting	3.039
72	Hf	$5s$	0	Pseudopotential Fitting	1.338
		$6s$	0	Orthogonalization	1.338
		$5p$	1	Pseudopotential Fitting	3.698
		$5d$	2	Pseudopotential Fitting	4.275
		$6p$	1	Orthogonalization	3.698
73	Ta	$5s$	0	Pseudopotential Fitting	1.377
		$6s$	0	Orthogonalization	1.377
		$5p$	1	Pseudopotential Fitting	3.680
		$6p$	1	Orthogonalization	3.680
		$5d$	2	Pseudopotential Fitting	4.827
74	W	$5s$	0	Pseudopotential Fitting	1.443
		$6s$	0	Orthogonalization	1.443
		$5p$	1	Pseudopotential Fitting	3.786
		$6p$	1	Orthogonalization	3.786
		$5d$	2	Pseudopotential Fitting	5.147
75	Re	$5s$	0	Pseudopotential Fitting	1.473
		$6s$	0	Orthogonalization	1.473
		$5p$	1	Pseudopotential Fitting	3.874
		$6p$	1	Orthogonalization	3.874
		$5d$	2	Pseudopotential Fitting	5.504
76	Os	$5s$	0	Pseudopotential Fitting	1.534
		$6s$	0	Orthogonalization	1.534
		$5p$	1	Pseudopotential Fitting	4.074
		$6p$	1	Orthogonalization	4.074
		$5d$	2	Pseudopotential Fitting	5.950
77	Ir	$6s$	0	Pseudopotential Fitting	0.418
		$5p$	1	Pseudopotential Fitting	3.776
		$6p$	1	Orthogonalization	3.776
		$5d$	2	Pseudopotential Fitting	5.503
78	Pt	$6s$	0	Pseudopotential Fitting	0.436
		$5p$	1	Pseudopotential Fitting	3.993
		$6p$	1	Orthogonalization	3.993
		$5d$	2	Pseudopotential Fitting	5.852
79	Au	$5s$	0	Pseudopotential Fitting	1.840
		$6s$	0	Orthogonalization	1.840
		$5p$	1	Pseudopotential Fitting	4.682
		$5d$	2	Pseudopotential Fitting	6.697
		$6p$	1	Orthogonalization	4.682
80	Hg	$5s$	0	Pseudopotential Fitting	1.891
		$6s$	0	Orthogonalization	1.891
		$5p$	1	Pseudopotential Fitting	4.840
		$5d$	2	Pseudopotential Fitting	6.962
		$6p$	1	Orthogonalization	4.840
81	Tl	$6s$	0	Pseudopotential Fitting	0.548
		$6p$	1	Pseudopotential Fitting	1.219
		$5d$	2	Pseudopotential Fitting	4.322
82	Pb	$6s$	0	Pseudopotential Fitting	0.607
		$6p$	1	Pseudopotential Fitting	1.477
		$5d$	2	Pseudopotential Fitting	5.953
83	Bi	$6s$	0	Pseudopotential Fitting	0.632
		$6p$	1	Pseudopotential Fitting	1.638
		$5d$	2	Pseudopotential Fitting	4.464
84	Po	$6s$	0	Pseudopotential Fitting	0.622
		$6p$	1	Pseudopotential Fitting	1.658
		$5d$	2	Pseudopotential Fitting	7.897
86	Rn	$6s$	0	Pseudopotential Fitting	0.615
		$6p$	1	Pseudopotential Fitting	1.915
		$5d$	2	Pseudopotential Fitting	8.335

SUPPLEMENTARY TABLE V: Values of α and minimal set of orbitals for the pseudopotentials of the PseudoDojo norm-conserving fully-relativistic v0.4 standard library with orthonormalization fitting.

Z	Element	Orbital	l	j	Source	$\alpha(\text{bohr}^{-1})$
1	H	$1s$	0	0.5	Pseudopotential	—
2	He	$1s$	0	0.5	Pseudopotential	—
3	Li	$1s$	0	0.5	Pseudopotential	—
		$2s$	0	0.5	Pseudopotential	—
		$2p$	1	0.5	OpenMX Fitting	1.114
		$2p$	1	1.5	OpenMX Fitting	1.114
4	Be	$1s$	0	0.5	Pseudopotential	—
		$2s$	0	0.5	Pseudopotential	—
		$2p$	1	0.5	OpenMX Fitting	1.834
		$2p$	1	1.5	OpenMX Fitting	1.834
5	B	$2s$	0	0.5	Pseudopotential	—
		$2p$	1	0.5	Pseudopotential	—
		$2p$	1	1.5	Pseudopotential	—
6	C	$2s$	0	0.5	Pseudopotential	—
		$2p$	1	0.5	Pseudopotential	—
		$2p$	1	1.5	Pseudopotential	—
7	N	$2s$	0	0.5	Pseudopotential	—
		$2p$	1	0.5	Pseudopotential	—
		$2p$	1	1.5	Pseudopotential	—
8	O	$2s$	0	0.5	Pseudopotential	—
		$2p$	1	0.5	Pseudopotential	—
		$2p$	1	1.5	Pseudopotential	—
9	F	$2s$	0	0.5	Pseudopotential	—
		$2p$	1	0.5	Pseudopotential	—
		$2p$	1	1.5	Pseudopotential	—
10	Ne	$2s$	0	0.5	Pseudopotential	—
		$2p$	1	0.5	Pseudopotential	—
		$2p$	1	1.5	Pseudopotential	—
11	Na	$2s$	0	0.5	Pseudopotential	—
		$3s$	0	0.5	Pseudopotential	—
		$2p$	1	0.5	Pseudopotential	—
		$2p$	1	1.5	Pseudopotential	—
		$3p$	1	0.5	Orthogonalization	5.600
		$3p$	1	1.5	Orthogonalization	5.600
12	Mg	$2s$	0	0.5	Pseudopotential	—
		$3s$	0	0.5	Pseudopotential	—
		$2p$	1	0.5	Pseudopotential	—
		$2p$	1	1.5	Pseudopotential	—
		$3p$	1	0.5	Orthogonalization	6.500
		$3p$	1	1.5	Orthogonalization	6.400
13	Al	$3s$	0	0.5	Pseudopotential	—
		$3p$	1	0.5	Pseudopotential	—
		$3p$	1	1.5	Pseudopotential	—
14	Si	$3s$	0	0.5	Pseudopotential	—
		$3p$	1	0.5	Pseudopotential	—
		$3p$	1	1.5	Pseudopotential	—
15	P	$3s$	0	0.5	Pseudopotential	—
		$3p$	1	0.5	Pseudopotential	—
		$3p$	1	1.5	Pseudopotential	—
16	S	$3s$	0	0.5	Pseudopotential	—
		$3p$	1	0.5	Pseudopotential	—
		$3p$	1	1.5	Pseudopotential	—
17	Cl	$3s$	0	0.5	Pseudopotential	—
		$3p$	1	0.5	Pseudopotential	—
		$3p$	1	1.5	Pseudopotential	—
18	Ar	$3s$	0	0.5	Pseudopotential	—
		$3p$	1	0.5	Pseudopotential	—
		$3p$	1	1.5	Pseudopotential	—
19	K	$3s$	0	0.5	Pseudopotential	—
		$4s$	0	0.5	Pseudopotential	—
		$3p$	1	0.5	Pseudopotential	—
		$3p$	1	1.5	Pseudopotential	—
		$3d$	2	1.5	OpenMX Fitting	2.727
		$3d$	2	2.5	OpenMX Fitting	2.727
20	Ca	$3s$	0	0.5	Pseudopotential	—
		$4s$	0	0.5	Pseudopotential	—
		$3p$	1	0.5	Pseudopotential	—
		$3p$	1	1.5	Pseudopotential	—
		$3d$	2	1.5	OpenMX Fitting	3.983
		$3d$	2	2.5	OpenMX Fitting	3.983
21	Sc	$3s$	0	0.5	Pseudopotential	—
		$4s$	0	0.5	Pseudopotential	—
		$3p$	1	0.5	Pseudopotential	—
		$3p$	1	1.5	Pseudopotential	—
		$3d$	2	1.5	Pseudopotential	—
		$3d$	2	2.5	Pseudopotential	—
		$4p$	1	0.5	Orthogonalization	4.400
		$4p$	1	1.5	Orthogonalization	4.300
22	Ti	$3s$	0	0.5	Pseudopotential	—
		$4s$	0	0.5	Pseudopotential	—
		$3p$	1	0.5	Pseudopotential	—
		$3p$	1	1.5	Pseudopotential	—
		$3d$	2	1.5	Pseudopotential	—
		$3d$	2	2.5	Pseudopotential	—
		$4p$	1	0.5	Orthogonalization	4.700
		$4p$	1	1.5	Orthogonalization	4.700
23	V	$3s$	0	0.5	Pseudopotential	—
		$4s$	0	0.5	Pseudopotential	—
		$3p$	1	0.5	Pseudopotential	—
		$3p$	1	1.5	Pseudopotential	—
		$3d$	2	1.5	Pseudopotential	—
		$3d$	2	2.5	Pseudopotential	—
		$4p$	1	0.5	Orthogonalization	5.000
		$4p$	1	1.5	Orthogonalization	5.000
24	Cr	$3s$	0	0.5	Pseudopotential	—
		$4s$	0	0.5	Pseudopotential	—
		$3p$	1	0.5	Pseudopotential	—
		$3p$	1	1.5	Pseudopotential	—
		$3d$	2	1.5	Pseudopotential	—
		$3d$	2	2.5	Pseudopotential	—
		$4p$	1	0.5	Orthogonalization	5.300
		$4p$	1	1.5	Orthogonalization	5.200
25	Mn	$3s$	0	0.5	Pseudopotential	—
		$4s$	0	0.5	Pseudopotential	—
		$3p$	1	0.5	Pseudopotential	—
		$3p$	1	1.5	Pseudopotential	—
		$3d$	2	1.5	Pseudopotential	—
		$3d$	2	2.5	Pseudopotential	—
		$4p$	1	0.5	Orthogonalization	5.600
		$4p$	1	1.5	Orthogonalization	5.600
26	Fe	$3s$	0	0.5	Pseudopotential	—
		$4s$	0	0.5	Pseudopotential	—
		$3p$	1	0.5	Pseudopotential	—
		$3p$	1	1.5	Pseudopotential	—
		$3d$	2	1.5	Pseudopotential	—
		$3d$	2	2.5	Pseudopotential	—
		$4p$	1	0.5	Orthogonalization	6.000
		$4p$	1	1.5	Orthogonalization	5.900

Z	Element	Orbital	l	j	Source	α(bohr^{-1})
27	Co	$3s$	0	0.5	Pseudopotential	—
		$4s$	0	0.5	Pseudopotential	—
		$3p$	1	0.5	Pseudopotential	—
		$3p$	1	1.5	Pseudopotential	—
		$3d$	2	1.5	Pseudopotential	—
		$3d$	2	2.5	Pseudopotential	—
		$4p$	1	0.5	Orthogonalization	6.300
		$4p$	1	1.5	Orthogonalization	6.200
28	Ni	$3s$	0	0.5	Pseudopotential	—
		$4s$	0	0.5	Pseudopotential	—
		$3p$	1	0.5	Pseudopotential	—
		$3p$	1	1.5	Pseudopotential	—
		$3d$	2	1.5	Pseudopotential	—
		$3d$	2	2.5	Pseudopotential	—
		$4p$	1	0.5	Orthogonalization	6.500
		$4p$	1	1.5	Orthogonalization	6.500
29	Cu	$3s$	0	0.5	Pseudopotential	—
		$4s$	0	0.5	Pseudopotential	—
		$3p$	1	0.5	Pseudopotential	—
		$3p$	1	1.5	Pseudopotential	—
		$3d$	2	1.5	Pseudopotential	—
		$3d$	2	2.5	Pseudopotential	—
		$4p$	1	0.5	Orthogonalization	6.700
		$4p$	1	1.5	Orthogonalization	6.600
30	Zn	$3s$	0	0.5	Pseudopotential	—
		$4s$	0	0.5	Pseudopotential	—
		$3p$	1	0.5	Pseudopotential	—
		$3p$	1	1.5	Pseudopotential	—
		$3d$	2	1.5	Pseudopotential	—
		$3d$	2	2.5	Pseudopotential	—
		$4p$	1	0.5	Orthogonalization	6.900
		$4p$	1	1.5	Orthogonalization	6.800
31	Ga	$4s$	0	0.5	Pseudopotential	—
		$4p$	1	0.5	Pseudopotential	—
		$4p$	1	1.5	Pseudopotential	—
		$3d$	2	1.5	Pseudopotential	—
		$3d$	2	2.5	Pseudopotential	—
32	Ge	$4s$	0	0.5	Pseudopotential	—
		$4p$	1	0.5	Pseudopotential	—
		$4p$	1	1.5	Pseudopotential	—
		$3d$	2	1.5	Pseudopotential	—
		$3d$	2	2.5	Pseudopotential	—
33	As	$4s$	0	0.5	Pseudopotential	—
		$4p$	1	0.5	Pseudopotential	—
		$4p$	1	1.5	Pseudopotential	—
		$3d$	2	1.5	Pseudopotential	—
		$3d$	2	2.5	Pseudopotential	—
34	Se	$4s$	0	0.5	Pseudopotential	—
		$4p$	1	0.5	Pseudopotential	—
		$4p$	1	1.5	Pseudopotential	—
		$3d$	2	1.5	Pseudopotential	—
		$3d$	2	2.5	Pseudopotential	—
35	Br	$4s$	0	0.5	Pseudopotential	—
		$4p$	1	0.5	Pseudopotential	—
		$4p$	1	1.5	Pseudopotential	—
36	Kr	$4s$	0	0.5	Pseudopotential	—
		$4p$	1	0.5	Pseudopotential	—
		$4p$	1	1.5	Pseudopotential	—

Z	Element	Orbital	l	j	Source	α(bohr^{-1})
37	Rb	$4s$	0	0.5	Pseudopotential	—
		$5s$	0	0.5	Pseudopotential	—
		$4p$	1	0.5	Pseudopotential	—
		$4p$	1	1.5	Pseudopotential	—
		$4d$	2	1.5	OpenMX Fitting	2.197
		$4d$	2	2.5	OpenMX Fitting	2.197
38	Sr	$4s$	0	0.5	Pseudopotential	—
		$5s$	0	0.5	Pseudopotential	—
		$4p$	1	0.5	Pseudopotential	—
		$4p$	1	1.5	Pseudopotential	—
		$4d$	2	1.5	OpenMX Fitting	4.400
		$4d$	2	2.5	OpenMX Fitting	4.400
39	Y	$4s$	0	0.5	Pseudopotential	—
		$5s$	0	0.5	Pseudopotential	—
		$4p$	1	0.5	Pseudopotential	—
		$4p$	1	1.5	Pseudopotential	—
		$4d$	2	1.5	Pseudopotential	—
		$4d$	2	2.5	Pseudopotential	—
		$5p$	1	0.5	Orthogonalization	3.600
		$5p$	1	1.5	Orthogonalization	3.500
40	Zr	$4s$	0	0.5	Pseudopotential	—
		$5s$	0	0.5	Pseudopotential	—
		$4p$	1	0.5	Pseudopotential	—
		$4p$	1	1.5	Pseudopotential	—
		$4d$	2	1.5	Pseudopotential	—
		$4d$	2	2.5	Pseudopotential	—
		$5p$	1	0.5	Orthogonalization	3.900
		$5p$	1	1.5	Orthogonalization	3.800
41	Nb	$4s$	0	0.5	Pseudopotential	—
		$5s$	0	0.5	Pseudopotential	—
		$4p$	1	0.5	Pseudopotential	—
		$4p$	1	1.5	Pseudopotential	—
		$4d$	2	1.5	Pseudopotential	—
		$4d$	2	2.5	Pseudopotential	—
		$5p$	1	0.5	Orthogonalization	4.000
		$5p$	1	1.5	Orthogonalization	3.900
42	Mo	$4s$	0	0.5	Pseudopotential	—
		$5s$	0	0.5	Pseudopotential	—
		$4p$	1	0.5	Pseudopotential	—
		$4p$	1	1.5	Pseudopotential	—
		$4d$	2	1.5	Pseudopotential	—
		$4d$	2	2.5	Pseudopotential	—
		$5p$	1	0.5	Orthogonalization	4.300
		$5p$	1	1.5	Orthogonalization	4.200
43	Tc	$4s$	0	0.5	Pseudopotential	—
		$5s$	0	0.5	Pseudopotential	—
		$4p$	1	0.5	Pseudopotential	—
		$4p$	1	1.5	Pseudopotential	—
		$4d$	2	1.5	Pseudopotential	—
		$4d$	2	2.5	Pseudopotential	—
		$5p$	1	0.5	Orthogonalization	4.500
		$5p$	1	1.5	Orthogonalization	4.400
44	Ru	$4s$	0	0.5	Pseudopotential	—
		$5s$	0	0.5	Pseudopotential	—
		$4p$	1	0.5	Pseudopotential	—
		$4p$	1	1.5	Pseudopotential	—
		$4d$	2	1.5	Pseudopotential	—
		$4d$	2	2.5	Pseudopotential	—
		$5p$	1	0.5	Orthogonalization	4.700
		$5p$	1	1.5	Orthogonalization	4.600

Z Element	Orbital	l	j	Source	α(bohr^{-1})
45 Rh	4s	0	0.5	Pseudopotential	—
	5s	0	0.5	Pseudopotential	—
	4p	1	0.5	Pseudopotential	—
	4p	1	1.5	Pseudopotential	—
	4d	2	1.5	Pseudopotential	—
	4d	2	2.5	Pseudopotential	—
	5p	1	0.5	Orthogonalization	4.900
	5p	1	1.5	Orthogonalization	4.800
46 Pd	4s	0	0.5	Pseudopotential	—
	4p	1	0.5	Pseudopotential	—
	4p	1	1.5	Pseudopotential	—
	4d	2	1.5	Pseudopotential	—
	4d	2	2.5	Pseudopotential	—
	5s	0	0.5	Orthogonalization	1.900
	5p	1	0.5	Orthogonalization	5.100
	5p	1	1.5	Orthogonalization	4.900
47 Ag	4s	0	0.5	Pseudopotential	—
	5s	0	0.5	Pseudopotential	—
	4p	1	0.5	Pseudopotential	—
	4p	1	1.5	Pseudopotential	—
	4d	2	1.5	Pseudopotential	—
	4d	2	2.5	Pseudopotential	—
	5p	1	0.5	Orthogonalization	5.400
	5p	1	1.5	Orthogonalization	5.200
48 Cd	4s	0	0.5	Pseudopotential	—
	5s	0	0.5	Pseudopotential	—
	4p	1	0.5	Pseudopotential	—
	4p	1	1.5	Pseudopotential	—
	4d	2	1.5	Pseudopotential	—
	4d	2	2.5	Pseudopotential	—
	5p	1	0.5	Orthogonalization	5.600
	5p	1	1.5	Orthogonalization	5.400
49 In	5s	0	0.5	Pseudopotential	—
	5p	1	0.5	Pseudopotential	—
	5p	1	1.5	Pseudopotential	—
	4d	2	1.5	Pseudopotential	—
	4d	2	2.5	Pseudopotential	—
50 Sn	5s	0	0.5	Pseudopotential	—
	5p	1	0.5	Pseudopotential	—
	5p	1	1.5	Pseudopotential	—
	4d	2	1.5	Pseudopotential	—
	4d	2	2.5	Pseudopotential	—
51 Sb	5s	0	0.5	Pseudopotential	—
	5p	1	0.5	Pseudopotential	—
	5p	1	1.5	Pseudopotential	—
	4d	2	1.5	Pseudopotential	—
	4d	2	2.5	Pseudopotential	—
52 Te	5s	0	0.5	Pseudopotential	—
	5p	1	0.5	Pseudopotential	—
	5p	1	1.5	Pseudopotential	—
	4d	2	1.5	Pseudopotential	—
	4d	2	2.5	Pseudopotential	—
53 I	5s	0	0.5	Pseudopotential	—
	5p	1	0.5	Pseudopotential	—
	5p	1	1.5	Pseudopotential	—
54 Xe	5s	0	0.5	Pseudopotential	—
	5p	1	0.5	Pseudopotential	—
	5p	1	1.5	Pseudopotential	—
55 Cs	5s	0	0.5	Pseudopotential	—
	6s	0	0.5	Pseudopotential	—
	5p	1	0.5	Pseudopotential	—
	5p	1	1.5	Pseudopotential	—
	5d	2	1.5	OpenMX Fitting	3.566
	5d	2	2.5	OpenMX Fitting	3.566
56 Ba	5s	0	0.5	Pseudopotential	—
	6s	0	0.5	Pseudopotential	—
	5p	1	0.5	Pseudopotential	—
	5p	1	1.5	Pseudopotential	—
	5d	2	1.5	OpenMX Fitting	3.039
	5d	2	2.5	OpenMX Fitting	3.039
72 Hf	5s	0	0.5	Pseudopotential	—
	6s	0	0.5	Pseudopotential	—
	5p	1	0.5	Pseudopotential	—
	5p	1	1.5	Pseudopotential	—
	5d	2	1.5	Pseudopotential	—
	5d	2	2.5	Pseudopotential	—
	6p	1	0.5	Orthogonalization	4.200
	6p	1	1.5	Orthogonalization	3.800
73 Ta	5s	0	0.5	Pseudopotential	—
	6s	0	0.5	Pseudopotential	—
	5p	1	0.5	Pseudopotential	—
	5p	1	1.5	Pseudopotential	—
	5d	2	1.5	Pseudopotential	—
	5d	2	2.5	Pseudopotential	—
	6p	1	0.5	Orthogonalization	4.300
	6p	1	1.5	Orthogonalization	4.000
74 W	5s	0	0.5	Pseudopotential	—
	6s	0	0.5	Pseudopotential	—
	5p	1	0.5	Pseudopotential	—
	5p	1	1.5	Pseudopotential	—
	5d	2	1.5	Pseudopotential	—
	5d	2	2.5	Pseudopotential	—
	6p	1	0.5	Orthogonalization	4.500
	6p	1	1.5	Orthogonalization	4.100
75 Re	5s	0	0.5	Pseudopotential	—
	6s	0	0.5	Pseudopotential	—
	5p	1	0.5	Pseudopotential	—
	5p	1	1.5	Pseudopotential	—
	5d	2	1.5	Pseudopotential	—
	5d	2	2.5	Pseudopotential	—
	6p	1	0.5	Orthogonalization	4.700
	6p	1	1.5	Orthogonalization	4.300
76 Os	5s	0	0.5	Pseudopotential	—
	6s	0	0.5	Pseudopotential	—
	5p	1	0.5	Pseudopotential	—
	5p	1	1.5	Pseudopotential	—
	5d	2	1.5	Pseudopotential	—
	5d	2	2.5	Pseudopotential	—
	6p	1	0.5	Orthogonalization	4.800
	6p	1	1.5	Orthogonalization	4.400
77 Ir	5s	0	0.5	Pseudopotential	—
	6s	0	0.5	Pseudopotential	—
	5p	1	0.5	Pseudopotential	—
	5p	1	1.5	Pseudopotential	—
	5d	2	1.5	Pseudopotential	—
	5d	2	2.5	Pseudopotential	—
	6p	1	0.5	Orthogonalization	5.000
	6p	1	1.5	Orthogonalization	4.600

Z Element	Orbital	l	j	Source	α(bohr^{-1})
78 Pt	$5s$	0	0.5	Pseudopotential	—
	$6s$	0	0.5	Pseudopotential	—
	$5p$	1	0.5	Pseudopotential	—
	$5p$	1	1.5	Pseudopotential	—
	$5d$	2	1.5	Pseudopotential	—
	$5d$	2	2.5	Pseudopotential	—
	$6p$	1	0.5	Orthogonalization	5.100
	$6p$	1	1.5	Orthogonalization	4.600
79 Au	$5s$	0	0.5	Pseudopotential	—
	$6s$	0	0.5	Pseudopotential	—
	$5p$	1	0.5	Pseudopotential	—
	$5p$	1	1.5	Pseudopotential	—
	$5d$	2	1.5	Pseudopotential	—
	$5d$	2	2.5	Pseudopotential	—
	$6p$	1	0.5	Orthogonalization	5.300
	$6p$	1	1.5	Orthogonalization	4.800
80 Hg	$5s$	0	0.5	Pseudopotential	—
	$6s$	0	0.5	Pseudopotential	—
	$5p$	1	0.5	Pseudopotential	—
	$5p$	1	1.5	Pseudopotential	—
	$5d$	2	1.5	Pseudopotential	—
	$5d$	2	2.5	Pseudopotential	—
	$6p$	1	0.5	Orthogonalization	5.500
	$6p$	1	1.5	Orthogonalization	5.000
81 Tl	$6s$	0	0.5	Pseudopotential	—
	$6p$	1	0.5	Pseudopotential	—
	$6p$	1	1.5	Pseudopotential	—
	$5d$	2	1.5	Pseudopotential	—
	$5d$	2	2.5	Pseudopotential	—
82 Pb	$6s$	0	0.5	Pseudopotential	—
	$6p$	1	0.5	Pseudopotential	—
	$6p$	1	1.5	Pseudopotential	—
	$5d$	2	1.5	Pseudopotential	—
	$5d$	2	2.5	Pseudopotential	—
83 Bi	$6s$	0	0.5	Pseudopotential	—
	$6p$	1	0.5	Pseudopotential	—
	$6p$	1	1.5	Pseudopotential	—
	$5d$	2	1.5	Pseudopotential	—
	$5d$	2	2.5	Pseudopotential	—
84 Po	$6s$	0	0.5	Pseudopotential	—
	$6p$	1	0.5	Pseudopotential	—
	$6p$	1	1.5	Pseudopotential	—
	$5d$	2	1.5	Pseudopotential	—
	$5d$	2	2.5	Pseudopotential	—
86 Rn	$6s$	0	0.5	Pseudopotential	—
	$6p$	1	0.5	Pseudopotential	—
	$6p$	1	1.5	Pseudopotential	—
	$5d$	2	1.5	Pseudopotential	—
	$5d$	2	2.5	Pseudopotential	—

SUPPLEMENTARY TABLE VI: Source of the pseudopotentials in the `modified-pslibrary` set. We started by selecting pseudopotentials based on the suggested list [4] from pslibrary. However, during DFT calculations, we encountered convergence errors with certain pseudopotentials. Thus, some pseudopotentials were replaced with either older versions from pslibrary or alternatives from pseudoDojo, as reported in this table.

Z	Elem.	Source	Filename
1	H	pslibrary	H.rel-pbe-kjpaw_psl.1.0.0.UPF
2	He	pslibrary	He.rel-pbe-kjpaw_psl.1.0.0.UPF
3	Li	pslibrary	Li.rel-pbe-sl-kjpaw_psl.1.0.0.UPF
4	Be	pslibrary	Be.rel-pbe-sl-kjpaw_psl.1.0.0.UPF
5	B	pslibrary	B.rel-pbe-n-kjpaw_psl.1.0.0.UPF
6	C	pslibrary	C.rel-pbe-n-kjpaw_psl.1.0.0.UPF
7	N	pslibrary	N.rel-pbe-n-kjpaw_psl.1.0.0.UPF
8	O	pslibrary	O.rel-pbe-n-kjpaw_psl.0.1.UPF
9	F	pslibrary	F.rel-pbe-n-kjpaw_psl.1.0.0.UPF
10	Ne	pslibrary	Ne.rel-pbe-n-kjpaw_psl.1.0.0.UPF
11	Na	pslibrary	Na.rel-pbe-spnl-kjpaw_psl.1.0.0.UPF
12	Mg	pslibrary	Mg.rel-pbe-spnl-kjpaw_psl.1.0.0.UPF
13	Al	pslibrary	Al.rel-pbe-nl-kjpaw_psl.1.0.0.UPF
14	Si	pslibrary	Si.rel-pbe-nl-kjpaw_psl.1.0.0.UPF
15	P	pslibrary	P.rel-pbe-nl-kjpaw_psl.1.0.0.UPF
16	S	pslibrary	S.rel-pbe-nl-kjpaw_psl.1.0.0.UPF
17	Cl	pslibrary	Cl.rel-pbe-nl-kjpaw_psl.1.0.0.UPF
18	Ar	pslibrary	Ar.rel-pbe-nl-kjpaw_psl.1.0.0.UPF
19	K	pslibrary	K.rel-pbe-spn-kjpaw_psl.1.0.0.UPF
20	Ca	pslibrary	Ca.rel-pbe-spn-kjpaw_psl.1.0.0.UPF
21	Sc	pslibrary	Sc.rel-pbe-spn-kjpaw_psl.1.0.0.UPF
22	Ti	pslibrary	Ti.rel-pbe-spn-kjpaw_psl.1.0.0.UPF
23	V	pslibrary	V.rel-pbe-spnl-kjpaw_psl.1.0.0.UPF
24	Cr	pslibrary	Cr.rel-pbe-spn-kjpaw_psl.1.0.0.UPF
25	Mn	pslibrary	Mn.rel-pbe-spn-kjpaw_psl.0.3.1.UPF
26	Fe	pslibrary	Fe.rel-pbe-n-kjpaw_psl.0.2.4.UPF
27	Co	pslibrary	Co.rel-pbe-spn-kjpaw_psl.0.3.1.UPF
28	Ni	pslibrary	Ni.rel-pbe-n-kjpaw_psl.0.1.UPF
29	Cu	pslibrary	Cu.rel-pbe-dn-kjpaw_psl.1.0.0.UPF
30	Zn	pslibrary	Zn.rel-pbe-dn-kjpaw_psl.1.0.0.UPF
31	Ga	pslibrary	Ga.rel-pbe-dnl-kjpaw_psl.1.0.0.UPF
32	Ge	pslibrary	Ge.rel-pbe-n-kjpaw_psl.1.0.0.UPF
33	As	pslibrary	As.rel-pbe-n-kjpaw_psl.1.0.0.UPF
34	Se	pslibrary	Se.rel-pbe-n-kjpaw_psl.1.0.0.UPF
35	Br	pslibrary	Br.rel-pbe-n-kjpaw_psl.1.0.0.UPF
36	Kr	pslibrary	Kr.rel-pbe-dn-kjpaw_psl.1.0.0.UPF
37	Rb	pslibrary	Rb.rel-pbe-spn-kjpaw_psl.1.0.0.UPF
38	Sr	pslibrary	Sr.rel-pbe-spn-kjpaw_psl.1.0.0.UPF
39	Y	pslibrary	Y.rel-pbe-spn-kjpaw_psl.1.0.0.UPF
40	Zr	pslibrary	Zr.rel-pbe-spn-kjpaw_psl.1.0.0.UPF
41	Nb	pslibrary	Nb.rel-pbe-spn-kjpaw_psl.1.0.0.UPF
42	Mo	pslibrary	Mo.rel-pbe-spn-kjpaw_psl.1.0.0.UPF
43	Tc	pslibrary	Tc.rel-pbe-spn-kjpaw_psl.0.3.0.UPF
44	Ru	pslibrary	Ru.rel-pbe-spn-kjpaw_psl.1.0.0.UPF
45	Rh	pseudoDojo	Rh.UPF
46	Pd	pslibrary	Pd.rel-pbe-n-kjpaw_psl.1.0.0.UPF
47	Ag	pslibrary	Ag.rel-pbe-n-kjpaw_psl.1.0.0.UPF
48	Cd	pslibrary	Cd.rel-pbe-dn-kjpaw_psl.0.3.1.UPF
49	In	pslibrary	In.rel-pbe-dn-kjpaw_psl.1.0.0.UPF
50	Sn	pslibrary	Sn.rel-pbe-dn-kjpaw_psl.1.0.0.UPF
51	Sb	pslibrary	Sb.rel-pbe-n-kjpaw_psl.1.0.0.UPF
52	Te	pslibrary	Te.rel-pbe-n-kjpaw_psl.1.0.0.UPF
53	I	pslibrary	I.rel-pbe-n-kjpaw_psl.1.0.0.UPF
54	Xe	pslibrary	Xe.rel-pbe-dn-kjpaw_psl.1.0.0.UPF
55	Cs	pseudoDojo	Cs.UPF

Z	Elem.	Source	Filename
56	Ba	pslibrary	Ba.rel-pbe-spn-kjpaw_psl.1.0.0.UPF
72	Hf	pslibrary	Hf.rel-pbe-spn-kjpaw_psl.1.0.0.UPF
73	Ta	pslibrary	Ta.rel-pbe-spn-kjpaw_psl.0.2.UPF
74	W	pslibrary	W.rel-pbe-spn-kjpaw_psl.1.0.1.UPF
75	Re	pslibrary	Re.rel-pbe-spn-kjpaw_psl.1.0.0.UPF
76	Os	pslibrary	Os.rel-pbe-spn-kjpaw_psl.1.0.0.UPF
77	Ir	pslibrary	Ir.rel-pbe-n-kjpaw_psl.0.2.3.UPF
78	Pt	pslibrary	Pt.rel-pbe-n-kjpaw_psl.1.0.0.UPF
79	Au	pslibrary	Au.rel-pbe-n-kjpaw_psl.1.0.0.UPF
80	Hg	pslibrary	Hg.rel-pbe-n-kjpaw_psl.1.0.0.UPF
81	Tl	pslibrary	Tl.rel-pbe-dn-kjpaw_psl.1.0.0.UPF
82	Pb	pslibrary	Pb.rel-pbe-dn-kjpaw_psl.0.2.2.UPF
83	Bi	pslibrary	Bi.rel-pbe-dn-kjpaw_psl.1.0.0.UPF
84	Po	pslibrary	Po.rel-pbe-dn-kjpaw_psl.1.0.0.UPF
85	At	pslibrary	At.rel-pbe-dn-kjpaw_psl.1.0.0.UPF
86	Rn	pslibrary	Rn.rel-pbe-dn-kjpaw_psl.1.0.0.UPF
87	Fr	pslibrary	Fr.rel-pbe-spdn-kjpaw_psl.1.0.0.UPF
88	Ra	pslibrary	Ra.rel-pbe-spdn-kjpaw_psl.1.0.0.UPF

SUPPLEMENTARY TABLE VII: Values of α and minimal set of orbitals for the pseudopotentials of the `modified-pslibrary` set with orthonormalization fitting.

Z	Element	Orbital	l	j	Source	α(bohr^{-1})
1	H	$1s$	0	0.5	Pseudopotential	—
2	He	$1s$	0	0.5	Pseudopotential	—
3	Li	$1s$	0	0.5	Pseudopotential	—
		$2s$	0	0.5	Pseudopotential	—
		$2p$	1	0.5	Pseudopotential	—
		$2p$	1	1.5	Pseudopotential	—
4	Be	$1s$	0	0.5	Pseudopotential	—
		$2s$	0	0.5	Pseudopotential	—
		$2p$	1	0.5	Pseudopotential	—
		$2p$	1	1.5	Pseudopotential	—
5	B	$2s$	0	0.5	Pseudopotential	—
		$2p$	1	0.5	Pseudopotential	—
		$2p$	1	1.5	Pseudopotential	—
6	C	$2s$	0	0.5	Pseudopotential	—
		$2p$	1	0.5	Pseudopotential	—
		$2p$	1	1.5	Pseudopotential	—
7	N	$2s$	0	0.5	Pseudopotential	—
		$2p$	1	0.5	Pseudopotential	—
		$2p$	1	1.5	Pseudopotential	—
8	O	$2s$	0	0.5	Pseudopotential	—
		$2p$	1	0.5	Pseudopotential	—
		$2p$	1	1.5	Pseudopotential	—
9	F	$2s$	0	0.5	Pseudopotential	—
		$2p$	1	0.5	Pseudopotential	—
		$2p$	1	1.5	Pseudopotential	—
10	Ne	$2s$	0	0.5	Pseudopotential	—
		$2p$	1	0.5	Pseudopotential	—
		$2p$	1	1.5	Pseudopotential	—
11	Na	$2s$	0	0.5	Pseudopotential	—
		$3s$	0	0.5	Pseudopotential	—
		$2p$	1	0.5	Pseudopotential	—
		$2p$	1	1.5	Pseudopotential	—
		$3p$	1	0.5	Orthogonalization	4.900
		$3p$	1	1.5	Orthogonalization	4.900
12	Mg	$2s$	0	0.5	Pseudopotential	—
		$3s$	0	0.5	Pseudopotential	—
		$2p$	1	0.5	Pseudopotential	—
		$2p$	1	1.5	Pseudopotential	—
		$3p$	1	0.5	Orthogonalization	5.500
		$3p$	1	1.5	Orthogonalization	5.500
13	Al	$3s$	0	0.5	Pseudopotential	—
		$3p$	1	0.5	Pseudopotential	—
		$3p$	1	1.5	Pseudopotential	—
14	Si	$3s$	0	0.5	Pseudopotential	—
		$3p$	1	0.5	Pseudopotential	—
		$3p$	1	1.5	Pseudopotential	—
15	P	$3s$	0	0.5	Pseudopotential	—
		$3p$	1	0.5	Pseudopotential	—
		$3p$	1	1.5	Pseudopotential	—
16	S	$3s$	0	0.5	Pseudopotential	—
		$3p$	1	0.5	Pseudopotential	—
		$3p$	1	1.5	Pseudopotential	—
17	Cl	$3s$	0	0.5	Pseudopotential	—
		$3p$	1	0.5	Pseudopotential	—
		$3p$	1	1.5	Pseudopotential	—
18	Ar	$3s$	0	0.5	Pseudopotential	—
		$3p$	1	0.5	Pseudopotential	—
		$3p$	1	1.5	Pseudopotential	—

Z	Element	Orbital	l	j	Source	α(bohr^{-1})
19	K	$3s$	0	0.5	Pseudopotential	—
		$4s$	0	0.5	Pseudopotential	—
		$3p$	1	0.5	Pseudopotential	—
		$4p$	1	0.5	Pseudopotential	—
		$3p$	1	1.5	Pseudopotential	—
		$4p$	1	1.5	Pseudopotential	—
		$3d$	2	1.5	OpenMX Fitting	2.727
		$3d$	2	2.5	OpenMX Fitting	2.727
20	Ca	$3s$	0	0.5	Pseudopotential	—
		$4s$	0	0.5	Pseudopotential	—
		$3p$	1	0.5	Pseudopotential	—
		$4p$	1	0.5	Pseudopotential	—
		$3p$	1	1.5	Pseudopotential	—
		$4p$	1	1.5	Pseudopotential	—
		$3d$	2	1.5	OpenMX Fitting	3.983
		$3d$	2	2.5	OpenMX Fitting	3.983
21	Sc	$3s$	0	0.5	Pseudopotential	—
		$4s$	0	0.5	Pseudopotential	—
		$3p$	1	0.5	Pseudopotential	—
		$3p$	1	1.5	Pseudopotential	—
		$3d$	2	1.5	Pseudopotential	—
		$3d$	2	2.5	Pseudopotential	—
		$4p$	1	0.5	Orthogonalization	4.400
		$4p$	1	1.5	Orthogonalization	4.400
22	Ti	$3s$	0	0.5	Pseudopotential	—
		$4s$	0	0.5	Pseudopotential	—
		$3p$	1	0.5	Pseudopotential	—
		$3p$	1	1.5	Pseudopotential	—
		$3d$	2	1.5	Pseudopotential	—
		$3d$	2	2.5	Pseudopotential	—
		$4p$	1	0.5	Orthogonalization	4.600
		$4p$	1	1.5	Orthogonalization	4.500
23	V	$3s$	0	0.5	Pseudopotential	—
		$4s$	0	0.5	Pseudopotential	—
		$3p$	1	0.5	Pseudopotential	—
		$3p$	1	1.5	Pseudopotential	—
		$3d$	2	1.5	Pseudopotential	—
		$3d$	2	2.5	Pseudopotential	—
		$4p$	1	0.5	Orthogonalization	5.000
		$4p$	1	1.5	Orthogonalization	4.900
24	Cr	$3s$	0	0.5	Pseudopotential	—
		$4s$	0	0.5	Pseudopotential	—
		$3p$	1	0.5	Pseudopotential	—
		$3p$	1	1.5	Pseudopotential	—
		$3d$	2	1.5	Pseudopotential	—
		$3d$	2	2.5	Pseudopotential	—
		$4p$	1	0.5	Orthogonalization	5.200
		$4p$	1	1.5	Orthogonalization	5.200
25	Mn	$3s$	0	0.5	Pseudopotential	—
		$4s$	0	0.5	Pseudopotential	—
		$3p$	1	0.5	Pseudopotential	—
		$3p$	1	1.5	Pseudopotential	—
		$3d$	2	1.5	Pseudopotential	—
		$3d$	2	2.5	Pseudopotential	—
		$4p$	1	0.5	Orthogonalization	4.700
		$4p$	1	1.5	Orthogonalization	4.700
26	Fe	$4s$	0	0.5	Pseudopotential	—
		$4p$	1	0.5	Pseudopotential	—
		$4p$	1	1.5	Pseudopotential	—
		$3d$	2	1.5	Pseudopotential	—
		$3d$	2	2.5	Pseudopotential	—

Z	Element	Orbital	l	j	Source	α(bohr^{-1})
27	Co	$3s$	0	0.5	Pseudopotential	—
		$4s$	0	0.5	Pseudopotential	—
		$3p$	1	0.5	Pseudopotential	—
		$3p$	1	1.5	Pseudopotential	—
		$3d$	2	1.5	Pseudopotential	—
		$3d$	2	2.5	Pseudopotential	—
		$4p$	1	0.5	Orthogonalization	5.900
		$4p$	1	1.5	Orthogonalization	5.900
28	Ni	$4s$	0	0.5	Pseudopotential	—
		$4p$	1	0.5	Pseudopotential	—
		$4p$	1	1.5	Pseudopotential	—
		$3d$	2	1.5	Pseudopotential	—
		$3d$	2	2.5	Pseudopotential	—
29	Cu	$4s$	0	0.5	Pseudopotential	—
		$4p$	1	0.5	Pseudopotential	—
		$4p$	1	1.5	Pseudopotential	—
		$3d$	2	1.5	Pseudopotential	—
		$3d$	2	2.5	Pseudopotential	—
30	Zn	$4s$	0	0.5	Pseudopotential	—
		$4p$	1	0.5	Pseudopotential	—
		$4p$	1	1.5	Pseudopotential	—
		$3d$	2	1.5	Pseudopotential	—
		$3d$	2	2.5	Pseudopotential	—
31	Ga	$4s$	0	0.5	Pseudopotential	—
		$4p$	1	0.5	Pseudopotential	—
		$4p$	1	1.5	Pseudopotential	—
		$3d$	2	1.5	Pseudopotential	—
		$3d$	2	2.5	Pseudopotential	—
32	Ge	$4s$	0	0.5	Pseudopotential	—
		$4p$	1	0.5	Pseudopotential	—
		$4p$	1	1.5	Pseudopotential	—
33	As	$4s$	0	0.5	Pseudopotential	—
		$4p$	1	0.5	Pseudopotential	—
		$4p$	1	1.5	Pseudopotential	—
34	Se	$4s$	0	0.5	Pseudopotential	—
		$4p$	1	0.5	Pseudopotential	—
		$4p$	1	1.5	Pseudopotential	—
35	Br	$4s$	0	0.5	Pseudopotential	—
		$4p$	1	0.5	Pseudopotential	—
		$4p$	1	1.5	Pseudopotential	—
36	Kr	$4s$	0	0.5	Pseudopotential	—
		$4p$	1	0.5	Pseudopotential	—
		$4p$	1	1.5	Pseudopotential	—
		$3d$	2	1.5	Pseudopotential	—
		$3d$	2	2.5	Pseudopotential	—
37	Rb	$4s$	0	0.5	Pseudopotential	—
		$5s$	0	0.5	Pseudopotential	—
		$4p$	1	0.5	Pseudopotential	—
		$5p$	1	0.5	Pseudopotential	—
		$4p$	1	1.5	Pseudopotential	—
		$5p$	1	1.5	Pseudopotential	—
		$4d$	2	1.5	OpenMX Fitting	2.197
		$4d$	2	2.5	OpenMX Fitting	2.197
38	Sr	$4s$	0	0.5	Pseudopotential	—
		$5s$	0	0.5	Pseudopotential	—
		$4p$	1	0.5	Pseudopotential	—
		$5p$	1	0.5	Pseudopotential	—
		$4p$	1	1.5	Pseudopotential	—
		$5p$	1	1.5	Pseudopotential	—
		$4d$	2	1.5	OpenMX Fitting	4.400
		$4d$	2	2.5	OpenMX Fitting	4.400

Z	Element	Orbital	l	j	Source	α(bohr^{-}1)
39	Y	$4s$	0	0.5	Pseudopotential	—
		$5s$	0	0.5	Pseudopotential	—
		$4p$	1	0.5	Pseudopotential	—
		$5p$	1	0.5	Pseudopotential	—
		$4p$	1	1.5	Pseudopotential	—
		$5p$	1	1.5	Pseudopotential	—
		$4d$	2	1.5	Pseudopotential	—
		$4d$	2	2.5	Pseudopotential	—
40	Zr	$4s$	0	0.5	Pseudopotential	—
		$5s$	0	0.5	Pseudopotential	—
		$4p$	1	0.5	Pseudopotential	—
		$5p$	1	0.5	Pseudopotential	—
		$4p$	1	1.5	Pseudopotential	—
		$5p$	1	1.5	Pseudopotential	—
		$4d$	2	1.5	Pseudopotential	—
		$4d$	2	2.5	Pseudopotential	—
41	Nb	$4s$	0	0.5	Pseudopotential	—
		$5s$	0	0.5	Pseudopotential	—
		$4p$	1	0.5	Pseudopotential	—
		$5p$	1	0.5	Pseudopotential	—
		$4p$	1	1.5	Pseudopotential	—
		$5p$	1	1.5	Pseudopotential	—
		$4d$	2	1.5	Pseudopotential	—
		$4d$	2	2.5	Pseudopotential	—
42	Mo	$4s$	0	0.5	Pseudopotential	—
		$5s$	0	0.5	Pseudopotential	—
		$4p$	1	0.5	Pseudopotential	—
		$4p$	1	1.5	Pseudopotential	—
		$4d$	2	1.5	Pseudopotential	—
		$4d$	2	2.5	Pseudopotential	—
		$5p$	1	0.5	Orthogonalization	4.500
		$5p$	1	1.5	Orthogonalization	4.400
43	Tc	$4s$	0	0.5	Pseudopotential	—
		$5s$	0	0.5	Pseudopotential	—
		$4p$	1	0.5	Pseudopotential	—
		$5p$	1	0.5	Pseudopotential	—
		$4p$	1	1.5	Pseudopotential	—
		$5p$	1	1.5	Pseudopotential	—
		$4d$	2	1.5	Pseudopotential	—
		$4d$	2	2.5	Pseudopotential	—
44	Ru	$4s$	0	0.5	Pseudopotential	—
		$5s$	0	0.5	Pseudopotential	—
		$4p$	1	0.5	Pseudopotential	—
		$4p$	1	1.5	Pseudopotential	—
		$4d$	2	1.5	Pseudopotential	—
		$4d$	2	2.5	Pseudopotential	—
		$5p$	1	0.5	Orthogonalization	4.900
		$5p$	1	1.5	Orthogonalization	4.800
45	Rh	$4s$	0	0.5	Pseudopotential	—
		$5s$	0	0.5	Pseudopotential	—
		$4p$	1	0.5	Pseudopotential	—
		$4p$	1	1.5	Pseudopotential	—
		$4d$	2	1.5	Pseudopotential	—
		$4d$	2	2.5	Pseudopotential	—
		$5p$	1	0.5	Orthogonalization	4.900
		$5p$	1	1.5	Orthogonalization	4.800
46	Pd	$5s$	0	0.5	Pseudopotential	—
		$5p$	1	0.5	Pseudopotential	—
		$5p$	1	1.5	Pseudopotential	—
		$4d$	2	1.5	Pseudopotential	—
		$4d$	2	2.5	Pseudopotential	—
47	Ag	$5s$	0	0.5	Pseudopotential	—
		$5p$	1	0.5	Pseudopotential	—
		$5p$	1	1.5	Pseudopotential	—
		$4d$	2	1.5	Pseudopotential	—
		$4d$	2	2.5	Pseudopotential	—
48	Cd	$5s$	0	0.5	Pseudopotential	—
		$5p$	1	0.5	Pseudopotential	—
		$5p$	1	1.5	Pseudopotential	—
		$4d$	2	1.5	Pseudopotential	—
		$4d$	2	2.5	Pseudopotential	—
49	In	$5s$	0	0.5	Pseudopotential	—
		$5p$	1	0.5	Pseudopotential	—
		$5p$	1	1.5	Pseudopotential	—
		$4d$	2	1.5	Pseudopotential	—
		$4d$	2	2.5	Pseudopotential	—
50	Sn	$5s$	0	0.5	Pseudopotential	—
		$5p$	1	0.5	Pseudopotential	—
		$5p$	1	1.5	Pseudopotential	—
		$4d$	2	1.5	Pseudopotential	—
		$4d$	2	2.5	Pseudopotential	—
51	Sb	$5s$	0	0.5	Pseudopotential	—
		$5p$	1	0.5	Pseudopotential	—
		$5p$	1	1.5	Pseudopotential	—
52	Te	$5s$	0	0.5	Pseudopotential	—
		$5p$	1	0.5	Pseudopotential	—
		$5p$	1	1.5	Pseudopotential	—
53	I	$5s$	0	0.5	Pseudopotential	—
		$5p$	1	0.5	Pseudopotential	—
		$5p$	1	1.5	Pseudopotential	—
54	Xe	$5s$	0	0.5	Pseudopotential	—
		$5p$	1	0.5	Pseudopotential	—
		$5p$	1	1.5	Pseudopotential	—
		$4d$	2	1.5	Pseudopotential	—
		$4d$	2	2.5	Pseudopotential	—
55	Cs	$5s$	0	0.5	Pseudopotential	—
		$6s$	0	0.5	Pseudopotential	—
		$5p$	1	0.5	Pseudopotential	—
		$5p$	1	1.5	Pseudopotential	—
		$5d$	2	1.5	OpenMX Fitting	3.566
		$5d$	2	2.5	OpenMX Fitting	3.566
56	Ba	$5s$	0	0.5	Pseudopotential	—
		$6s$	0	0.5	Pseudopotential	—
		$5p$	1	0.5	Pseudopotential	—
		$5p$	1	1.5	Pseudopotential	—
		$5d$	2	1.5	OpenMX Fitting	3.039
		$5d$	2	2.5	OpenMX Fitting	3.039
72	Hf	$5s$	0	0.5	Pseudopotential	—
		$6s$	0	0.5	Pseudopotential	—
		$5p$	1	0.5	Pseudopotential	—
		$5p$	1	1.5	Pseudopotential	—
		$5d$	2	1.5	Pseudopotential	—
		$5d$	2	2.5	Pseudopotential	—
		$6p$	1	0.5	Orthogonalization	4.400
		$6p$	1	1.5	Orthogonalization	3.900
73	Ta	$5s$	0	0.5	Pseudopotential	—
		$6s$	0	0.5	Pseudopotential	—
		$5p$	1	0.5	Pseudopotential	—
		$6p$	1	0.5	Pseudopotential	—
		$5p$	1	1.5	Pseudopotential	—
		$6p$	1	1.5	Pseudopotential	—
		$5d$	2	1.5	Pseudopotential	—
		$5d$	2	2.5	Pseudopotential	—

Z	Element	Orbital	l	j	Source	α(bohr^{-}1)
74	W	5s	0	0.5	Pseudopotential	—
		6s	0	0.5	Pseudopotential	—
		5p	1	0.5	Pseudopotential	—
		6p	1	0.5	Pseudopotential	—
		5p	1	1.5	Pseudopotential	—
		6p	1	1.5	Pseudopotential	—
		5d	2	1.5	Pseudopotential	—
		5d	2	2.5	Pseudopotential	—
75	Re	5s	0	0.5	Pseudopotential	—
		6s	0	0.5	Pseudopotential	—
		5p	1	0.5	Pseudopotential	—
		5p	1	1.5	Pseudopotential	—
		5d	2	1.5	Pseudopotential	—
		5d	2	2.5	Pseudopotential	—
		6p	1	0.5	Orthogonalization	4.900
		6p	1	1.5	Orthogonalization	4.500
76	Os	5s	0	0.5	Pseudopotential	—
		6s	0	0.5	Pseudopotential	—
		5p	1	0.5	Pseudopotential	—
		5p	1	1.5	Pseudopotential	—
		5d	2	1.5	Pseudopotential	—
		5d	2	2.5	Pseudopotential	—
		6p	1	0.5	Orthogonalization	5.100
		6p	1	1.5	Orthogonalization	4.600
77	Ir	6s	0	0.5	Pseudopotential	—
		6p	1	0.5	Pseudopotential	—
		6p	1	1.5	Pseudopotential	—
		5d	2	1.5	Pseudopotential	—
		5d	2	2.5	Pseudopotential	—
78	Pt	6s	0	0.5	Pseudopotential	—
		6p	1	0.5	Pseudopotential	—
		6p	1	1.5	Pseudopotential	—
		5d	2	1.5	Pseudopotential	—
		5d	2	2.5	Pseudopotential	—
79	Au	6s	0	0.5	Pseudopotential	—
		6p	1	0.5	Pseudopotential	—
		6p	1	1.5	Pseudopotential	—
		5d	2	1.5	Pseudopotential	—
		5d	2	2.5	Pseudopotential	—
80	Hg	6s	0	0.5	Pseudopotential	—
		6p	1	0.5	Pseudopotential	—
		6p	1	1.5	Pseudopotential	—
		5d	2	1.5	Pseudopotential	—
		5d	2	2.5	Pseudopotential	—
81	Tl	6s	0	0.5	Pseudopotential	—
		6p	1	0.5	Pseudopotential	—
		6p	1	1.5	Pseudopotential	—
		5d	2	1.5	Pseudopotential	—
		5d	2	2.5	Pseudopotential	—
82	Pb	6s	0	0.5	Pseudopotential	—
		6p	1	0.5	Pseudopotential	—
		6p	1	1.5	Pseudopotential	—
		5d	2	1.5	Pseudopotential	—
		5d	2	2.5	Pseudopotential	—
83	Bi	6s	0	0.5	Pseudopotential	—
		6p	1	0.5	Pseudopotential	—
		6p	1	1.5	Pseudopotential	—
		5d	2	1.5	Pseudopotential	—
		5d	2	2.5	Pseudopotential	—
84	Po	6s	0	0.5	Pseudopotential	—
		6p	1	0.5	Pseudopotential	—
		6p	1	1.5	Pseudopotential	—
		5d	2	1.5	Pseudopotential	—
		5d	2	2.5	Pseudopotential	—
85	At	6s	0	0.5	Pseudopotential	—
		6p	1	0.5	Pseudopotential	—
		6p	1	1.5	Pseudopotential	—
		5d	2	1.5	Pseudopotential	—
		5d	2	2.5	Pseudopotential	—
86	Rn	6s	0	0.5	Pseudopotential	—
		6p	1	0.5	Pseudopotential	—
		6p	1	1.5	Pseudopotential	—
		5d	2	1.5	Pseudopotential	—
		5d	2	2.5	Pseudopotential	—
87	Fr	6s	0	0.5	Pseudopotential	—
		7s	0	0.5	Pseudopotential	—
		6p	1	0.5	Pseudopotential	—
		6p	1	1.5	Pseudopotential	—
		5d	2	1.5	Pseudopotential	—
		5d	2	2.5	Pseudopotential	—
88	Ra	6s	0	0.5	Pseudopotential	—
		7s	0	0.5	Pseudopotential	—
		6p	1	0.5	Pseudopotential	—
		6p	1	1.5	Pseudopotential	—
		5d	2	1.5	Pseudopotential	—
		5d	2	2.5	Pseudopotential	—

SUPPLEMENTARY TABLE VIII: Values of α and minimal set of orbitals for projectors from `OpenMX` fitting. We note that we only report values for those orbitals for which a value was fitted from OpenMX in this work (i.e., for orbitals that were not included in the PAOs for at least one pseudopotential family, and that did not have underlying orbitals with the same angular character, so that no orthogonality condition on the radial part could be enforced).

Z	Element	Filename	Orbital	l	α(bohr^{-1})
1	H	H5.0.pao	1s	0	1.075
2	He	He10.0.pao	1s	0	1.529
3	Li	Li12.0.pao	2s	0	2.520
			2p	1	1.114
4	Be	Be8.0.pao	2s	0	0.649
			2p	1	1.834
5	B	B9.0.pao	2s	0	0.833
			2p	1	2.143
6	C	C6.0.pao	2s	0	1.000
			2p	1	2.684
7	N	N6.0p.pao	2s	0	1.227
			2p	1	3.228
8	O	O6.0.pao	2s	0	1.412
			2p	1	3.755
9	F	F7.0.pao	2s	0	1.608
			2p	1	4.230
10	Ne	Ne11.0.pao	2s	0	1.797
			2p	1	4.748
11	Na	Na11.0.pao	3s	0	2.072
			3p	1	5.830
12	Mg	Mg7.0.pao	3s	0	0.587
			3p	1	6.876
13	Al	Al7.0.pao	3s	0	0.682
			3p	1	1.768
14	Si	Si8.0.pao	3s	0	0.765
			3p	1	1.876
15	P	P8.0.pao	3s	0	0.886
			3p	1	2.191
16	S	S9.0.pao	3s	0	0.992
			3p	1	2.432
17	Cl	Cl7.0.pao	3s	0	1.096
			3p	1	2.733
18	Ar	Ar11.0.pao	3s	0	1.197
			3p	1	3.001
19	K	K12.0.pao	4s	0	1.336
			3d	2	2.727
20	Ca	Ca9.0.pao	4s	0	1.474
			3d	2	3.983
21	Sc	Sc11.0.pao	4s	0	1.589
			4p	1	4.353
			3d	2	5.200
22	Ti	Ti11.0.pao	4s	0	1.698
			4p	1	4.628
			3d	2	5.246
23	V	V8.0.pao	4s	0	1.796
			4p	1	4.925
			3d	2	5.774
24	Cr	Cr6.0.pao	4s	0	1.902
			4p	1	5.222
			3d	2	6.565
25	Mn	Mn8.0.pao	4s	0	1.999
			4p	1	5.603
			3d	2	8.113
26	Fe	Fe6.0S.pao	4s	0	0.641
			4p	1	5.882
			3d	2	8.140
27	Co	Co8.0H.pao	4s	0	2.195
			4p	1	6.180
			3d	2	8.453
28	Ni	Ni10.0H.pao	4s	0	2.321
			4p	1	6.481
			3d	2	8.893
29	Cu	Cu8.0S.pao	4s	0	0.601
			4p	1	2.149
			3d	2	9.296
30	Zn	Zn10.0S.pao	4s	0	0.628
			4p	1	1.361
			3d	2	10.651
31	Ga	Ga7.0.pao	4s	0	0.729
			4p	1	1.781
32	Ge	Ge9.0.pao	4s	0	0.798
			4p	1	1.815
33	As	As9.0.pao	4s	0	0.875
			4p	1	2.035
34	Se	Se7.0.pao	4s	0	0.947
			4p	1	2.272
35	Br	Br7.0.pao	4s	0	1.018
			4p	1	2.447
36	Kr	Kr10.0.pao	4s	0	1.087
			4p	1	2.612
37	Rb	Rb13.0.pao	5s	0	1.182
			4d	2	2.197
38	Sr	Sr12.0.pao	5s	0	1.275
			4d	2	4.400
39	Y	Y10.0.pao	5s	0	1.348
			5p	1	3.560
			4d	2	3.758
40	Zr	Zr9.0.pao	5s	0	1.430
			5p	1	3.760
			4d	2	4.062
41	Nb	Nb9.0.pao	5s	0	1.505
			5p	1	3.981
			4d	2	4.590
42	Mo	Mo7.0.pao	5s	0	1.567
			5p	1	4.190
			4d	2	5.150
43	Tc	Tc11.0.pao	5s	0	1.641
			5p	1	4.411
			4d	2	5.645
44	Ru	Ru7.0.pao	5s	0	0.569
			5p	1	4.599
			4d	2	5.920
45	Rh	Rh9.0.pao	5s	0	0.534
			5p	1	4.800
			4d	2	6.305
46	Pd	Pd11.0.pao	5s	0	0.522
			5p	1	4.966
			4d	2	6.254
47	Ag	Ag9.0.pao	5s	0	0.547
			5p	1	5.191
			4d	2	7.107
48	Cd	Cd7.0.pao	5s	0	0.621
			5p	1	1.815
			4d	2	7.835

Z	Element	Filename	Orbital	l	α(bohr^{-1})
49	In	In7.0.pao	5s	0	0.668
			5p	1	1.684
50	Sn	Sn9.0.pao	5s	0	0.713
			5p	1	1.652
51	Sb	Sb11.0.pao	5s	0	0.771
			5p	1	1.791
52	Te	Te9.0.pao	5s	0	0.825
			5p	1	1.946
53	I	I7.0.pao	5s	0	0.879
			5p	1	2.144
54	Xe	Xe13.0.pao	5s	0	0.930
			5p	1	2.230
55	Cs	Cs12.0.pao	6s	0	1.001
			5d	2	3.566
56	Ba	Ba10.0.pao	6s	0	1.069
			5d	2	3.039
72	Hf	Hf11.0.pao	6s	0	1.544
			6p	1	3.885
			5d	2	4.081
73	Ta	Ta11.0.pao	6s	0	1.593
			6p	1	4.070
			5d	2	4.551
74	W	W7.0.pao	6s	0	0.577
			6p	1	4.137
			5d	2	4.789
75	Re	Re7.0.pao	6s	0	1.698
			6p	1	4.338
			5d	2	5.392

Z	Element	Filename	Orbital	l	α(bohr^{-1})
76	Os	Os7.0.pao	6s	0	0.600
			6p	1	4.490
			5d	2	5.623
77	Ir	Ir11.0.pao	6s	0	0.576
			6p	1	4.615
			5d	2	5.716
78	Pt	Pt9.0.pao	6s	0	0.590
			6p	1	4.758
			5d	2	5.988
79	Au	Au11.0.pao	6s	0	0.589
			6p	1	4.873
			5d	2	6.301
80	Hg	Hg8.0.pao	6s	0	0.638
			6p	1	5.073
			5d	2	6.837
81	Tl	Tl8.0.pao	6s	0	0.689
			6p	1	5.245
82	Pb	Pb8.0.pao	6s	0	0.742
			6p	1	1.666
83	Bi	Bi8.0.pao	6s	0	0.792
			6p	1	1.786
84	Po	Po10.0.pao	6s	0	0.839
			6p	1	1.850
86	Rn	Rn13.0.pao	6s	0	0.934
			6p	1	2.091

[1] J. Qiao, G. Pizzi, and N. Marzari, Projectability disentanglement for accurate and automated electronic-structure Hamiltonians, npj Computational Materials **9**, 208 (2023).
[2] S. V. Gallego, J. M. Perez-Mato, L. Elcoro, E. S. Tasci, R. M. Hanson, K. Momma, M. I. Aroyo, and G. Madariaga, MAGNDATA : towards a database of magnetic structures. I. The commensurate case, Journal of Applied Crystallography **49**, 1750 (2016).
[3] T. Ozaki, Variationally optimized atomic orbitals for large-scale electronic structures, Physical Review B **67**, 155108 (2003).
[4] Suggested pseudopotentials, `https://dalcorso.github.io/pslibrary/PP_list.html`, online; accessed 20 January 2025.
